# Application of the IASP Grading System to Identify Underlying Pain Mechanisms in Patients With Knee Osteoarthritis: A Prospective Cohort Study

**DOI:** 10.1097/AJP.0000000000001234

**Published:** 2024-07-17

**Authors:** Sophie Vervullens, Lotte Meert, Mira Meeus, Christiaan H.W. Heusdens, Peter Verdonk, Anthe Foubert, Emmanuel Abatih, Lies Durnez, Jonas Verbrugghe, Rob J.E.M. Smeets

**Affiliations:** * Research Group MOVANT, Department of Rehabilitation Sciences and Physiotherapy (REVAKI), University of Antwerp, Wilrijk, Belgium; † Research School CAPHRI, Department of Rehabilitation Medicine, Maastricht University, Maastricht, The Netherlands; ‡ Pain in Motion International Research Group (PiM), Antwerp, Belgium; § Department of Orthopedics and Traumatology, University Hospital of Antwerp, Antwerp; ‖ Faculty of Medicine and Health Sciences, University of Antwerp, Wilrijk; ¶ ORTHOCA, Antwerp, Belgium; # ASTARC Department, Antwerp University, Antwerp; ** Faculté des Sciences de la Motricité, Université catholique de Louvain, Louvain-La-Neuve; †† DASS (Center for Data Analysis and Statistical Science), Ghent University, Krijgslaan, Gent; ‡‡ REVAL-Rehabilitation Research Center, Faculty of Rehabilitation Sciences, Hasselt University, Hasselt, Belgium; §§ CIR Clinics in Revalidatie, location Eindhoven, The Netherlands

**Keywords:** knee osteoarthritis, pain mechanisms, nociplastic pain, total knee arthroplasty, precision medicine

## Abstract

**Objectives:**

This study aimed to apply the International Association for the Study of Pain (IASP) grading system for identifying nociplastic pain in knee osteoarthritis (KOA) awaiting total knee arthroplasty (TKA) and propose criteria to fine-tune decision-making. In addition, the study aimed to characterize a “probable” versus “no or possible” nociplastic pain mechanism using biopsychosocial variables and compare both groups in their 1-year post-TKA response.

**Methods:**

A secondary analysis of baseline data of a longitudinal prospective study involving 197 patients with KOA awaiting total TKA in Belgium and the Netherlands was performed. Two approaches, one considering 4 and the other 3 pain locations (step 2 of the grading system), were presented. Linear mixed model analyses were performed to compare the probable and no or possible nociplastic pain mechanism groups for several preoperative biopsychosocial-related variables and 1-year postoperative pain. Also, a sensitivity analysis, comparing 3 pain mechanism groups, was performed.

**Results:**

Thirty (15.22%—approach 4 pain locations) and 46 (23.35%–approach 3 pain locations) participants were categorized under probable nociplastic pain. Irrespective of the pain location approach or sensitivity analysis, the probable nociplastic pain group included more woman, was younger, exhibited worse results on various preoperative pain-related and psychological variables, and had more pain 1-year post-TKA compared with the other group.

**Discussion:**

This study proposed additional criteria to fine-tune the grading system for nociplastic pain (except for discrete/regional/multifocal/widespread pain) and characterized a subgroup of patients with KOA with probable nociplastic pain. Future research is warranted for further validation.

Knee osteoarthritis (KOA) is a heterogeneous condition in which different phenotypes (based on disease trajectory, clinical presentation, etiology, treatment response, etc.) are present, however, without clear consensus yet.^1,2^ Phenotypes based on clinical representation are considered as the most useful for optimizing treatment selection.^1,3^ To date, the experience of chronic pain is still the primary symptom of KOA and the main reason why individuals seek medical care.^4^ Moreover, approximately 20% or patients with KOA experience chronic pain after total knee arthroplasty (TKA).^5–7^ Because different mechanisms determine the pain perception, pain phenotyping in particular has become very important in this population.^8,9^

Previous research found that a subgroup of patients with KOA experiencing chronic pain presents with disturbed somatosensory functioning, which includes mechanisms of peripheral and central sensitization.^1,10–13^ This can be observed in humans as primary and secondary hyperalgesia and allodynia, respectively. Central sensitization is accompanied by disturbances in the brain, nociceptors, and facilitatory and inhibitory ascending and descending pathways of the central nervous system.^14^ Interestingly, a recent umbrella review also found evidence that preoperative disturbed somatosensory functioning can be associated with chronic post-TKA pain.^15^

Previous pain phenotyping research was mostly based on objective measurements and patient-reported outcome measurements (PROMS),^9^ but recently, also phenotyping based on pain mechanisms specifically has gained attention.^16^ The International Association for the Study of Pain (IASP) categorized musculoskeletal pain into 3 main pain mechanisms: nociceptive, neuropathic, or nociplastic (or a combination).^17,18^ Nociplastic pain is defined as “pain that arises from altered nociception despite no clear evidence of actual or threatened tissue damage causing the activation of peripheral nociceptors or evidence for disease or lesion of the somatosensory system causing the pain.”^17^ Moreover, having disproportionate pain, higher levels of negative psychological factors (pain catastrophizing, and anxiety), disturbed somatosensory functioning (enhanced temporal summation [TS]), and other comorbidities (eg, diabetes and heart disease) are thought to be related to nociplastic pain according to a Delphi consensus expert study^19^ and seem to be predictors for worse musculoskeletal pain prognosis or KOA treatment outcomes.^7,15,20,21^ Therefore, it is postulated that nociplastic pain mechanisms contribute to the knee pain and are (or have become) the predominant pain mechanism in at least a subgroup of patients with KOA.

Previous research has attempted to phenotype and characterize KOA or TKA patients according to pain mechanisms; however, this was restricted to comparing patients with KOA with nociceptive pain and neuropathic-like pain classified according to the (modified) PainDETECT questionnaire^22–24^ or did not focus on the “nociplastic” pain mechanism explicitly.^25^ Regarding the nociplastic pain mechanism, Shraim et al^19,26^ attempted to define a group of typical nociplastic pain characteristics based on the literature and a Delphi consensus expert study.

As such, to date, the identification of the predominant pain mechanism remains a challenge in research and clinical practice. Identifying the predominant pain mechanism and its response to treatment is expected to improve patient-tailored care,^27^ which aims to optimize treatment outcomes or slow disease progression by matching interventions to individuals’ specific characteristics.^28^ For example, studies found that patients with KOA with more neuropathic-like pain had worse long-term pain outcomes after TKA.^23,24^ Therefore, different or additional treatment approaches are advised depending on whether patients have a predominant nociplastic (central nervous system-targeted therapy such as cognitive-behavioral therapy, central-acting drugs, and pain neuroscience education), nociceptive (biomedical approach such as surgery, joint-targeted manual, or exercise therapy), or neuropathic pain mechanism (peripheral nerve-targeted drug, exercise, or manual therapy).^18,29,30^

Recently, a clinical decision tree grading system for identifying the nociplastic pain mechanism has also been proposed by IASP in collaboration with experts in this field.^18^ However, clear specific guidelines and cutoff scores are missing to decide whether the underlying pain mechanism is nociplastic or not. Hence, Kosek et al^18^ highlighted the importance of applying and validating their IASP grading system in specific chronic pain populations using clinically useful and reliable diagnostic tests. Detailed information about their original grading system can be found in that article ^18^ and the Materials and Methods section of this article.

This study aimed to investigate the application of the IASP grading system in KOA patients awaiting TKA, as well as to propose criteria and cutoffs specifically for KOA patients to fine-tune the decisions and different steps used in the grading system (AIM 1). In addition, existing differences regarding various preoperative biopsychosocial factors were compared between individuals categorized as having “no or possible” nociplastic pain compared with individuals having “probable” nociplastic pain following the grading system to further characterize groups^26^ (AIM 2). Finally, the response to TKA 1-year postsurgery was compared between groups using pain intensity scores (AIM 3).

## MATERIALS AND METHODS

This study consists of a secondary analysis of a multicenter longitudinal prospective study. The study is conducted by applying the Strengthening the Reporting of Observational Studies in Epidemiology (STROBE) statement.^31^

### Setting

The longitudinal prospective has been approved by the Ethical committees of the University Hospital of Antwerp and AZ Monica, Belgium (BE300201319366); and the academic Hospital of Maastricht and St. Jans Gasthuis Weert, the Netherlands (NL6465408618). The protocol was registered at ClinicalTrials.gov (NCT05380648). This study was already ongoing when the IASP grading system^18^ was published, but through data of this study many variables were covered, allowing to apply the IASP grading system to a cohort of KOA patients awaiting TKA (see paragraph “Different steps including methods to apply the IASP grading system in patients with KOA”). Preoperative data of this project, measured during March 2018 until July 2022, were used to answer AIM 1 and 2, whereas also the 1-year post-TKA pain score, measured during March 2019 and July 2023, was used to answer AIM 3.

### Participants

Patients with KOA were either approached and checked for eligibility in person at the orthopedic department in the Netherlands by a nurse, or via phone by one of the executive researchers (S.V. or L.M.) in Belgium. Eligibility criteria are presented in Table 1. After giving their consent to participate, participants completed questionnaires to gather demographic information and health-related characteristics on paper or online via Qualtrics (www.qualtrics.com). All participants were instructed to refrain from first-stage pain medication, coffee, and alcohol 24 hours before the physical measurements, which were conducted at the Sensoric Functioning Lab at the University of Antwerp’s campus “Drie Eiken” (Belgian participants) or at the orthopedic department of the academic Hospital of Maastricht and St. Jans Gasthuis Weert (Dutch participants) by 2 researchers (S.V. or L.M.). Both researchers followed a practical skills training and used the same measurement forms to ensure standardization.

**TABLE 1:** Eligibility Criteria Patients With Knee Osteoarthritis and Pain-free Controls

Inclusion criteria	Exclusion criteria
Patients with knee osteoarthritis	
Diagnosis of KOA ≥40 y old Awaiting primary TKA	Neurological, or systemic diseases possibly impacting pain (experiencing neuropathic-like pain symptoms according to patient interview part of DN-4, neurological diseases such as Parkinson, CVA, etc, and systemic diseases such as rheumatoid arthritis, polymyalgia rheumatica, cancer, etc).
Healthy participants	
Healthy adults ≥40 y old Free of current pain	Pain/discomfort in >3 body regions pain/discomfort (NRS ≥ 3) for >30 d during the past 12 months or at the moment of the testing NRS ≥ 3 (max 2/10) Pregnant women or women giving birth or breast feeding <1 y ago Having psychiatric, systemic, neurological or cardiovascular diseases Had radioor chemotherapy in the past Intake of opioids, antidepressants, anticonvulsant medication <2 wk before the test

CVA indicates cerebrovascular accident; DN, Douleur Neuropathique-4; KOA, knee osteoarthritis; NRS, numeric rating scale; TKA, total knee arthroplasty.

### AIM 1: Different Steps and Methods to Apply the IASP Grading System in Patients With KOA

**FIGURE 1:**
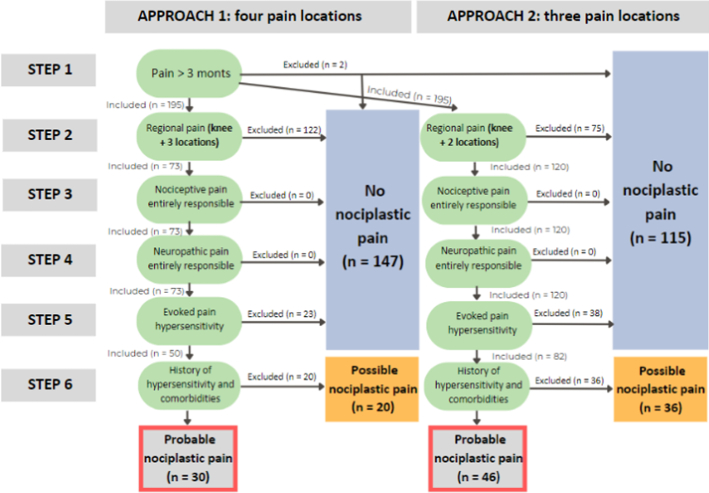
Flowchart of IASP grading system for having probable nociplastic pain.

All the steps of the IASP grading system with the chosen methods and interpretation used in the present KOA sample will be explained below and are also presented in Figure 1. Interpretation and methods were based on previous literature in KOA patients, other chronic MSK populations, and expert opinions.

#### Step 1: Chronic Pain (>3 Months)

The Knee Injury and Osteoarthritis Outcome Score (KOOS) subscale pain is a reliable and valid questionnaire^32^ and was used to define the presence of pain in the knee that would undergo TKA. This subscale consists of 9 questions of which the total score was transformed into a percentage score of 0 (indicating the worst pain) to 100 (indicating no pain).^33^ Roos^34^ defined a score of 87.5 or higher as “no pain.”^34^ This cutoff was chosen by Roos based on their previous findings regarding the “patient acceptable symptom state,” which identifies patients who are satisfied with their condition or not.^35^ The KOOS subscale pain does not include a question of “pain duration,” however, because KOA is still a chronic disease and all included participants were on the waiting list for a TKA—which is the last treatment strategy in the case of a nonresponse to conservative treatment strategies—^36^ sufficient arguments were available that the included sample of this study had >3 months pain if scores were below the cutoff presented by Roos.^34^

#### Step 2: Regional, Multifocal, or Widespread Rather than Discrete Pain

Participants had to indicate all body parts that they perceived as painful during the last week on a pain drawing showing a full body. The number of pain locations was counted and transferred to the digital database. Each large joint of the limbs (shoulder, elbow, wrist, hip, knee, and ankle, until the anatomical boundary of the joint), each finger and toe, a location between joints of limbs, cervical, thoracal, lumbar, sacroiliac or coccyx, head, jaw, and nose counted as one pain location (bilateral = 2 locations). As no specific cutoffs for separating regional, widespread, or multifocal pain from discrete pain were provided by Kosek et al,^18^ we followed different approaches.

##### Multifocal Approach 1

Previous research used a modified definition of widespread pain in a population with KOA: pain at the affected knee in addition to 3 other locations (4 locations in total).^37,38^ This modified definition was also used in current study for multifocal approach 1. All participants who fulfilled this modified definition fulfilled step 2.

##### Multifocal Approach 2

Because Kosek et al^18^ did not differentiate between the terms regional/widespread/multifocal, we decided to present a less stringent approach: participants had to report pain at the affected knee and at 2 additional pain locations (3 locations in total) to fulfill step 2.

##### Rather Discrete Pain

If participants reported pain at the knee with only one other location, the participant was categorized as having rather discrete pain. This approach was chosen because patients with KOA frequently experience bilateral KOA,^39,40^ thus eliminating the risk of classifying these patients as having nondiscrete pain. As such, these participants were categorized as having unlikely nociplastic pain.

The next steps will as such be evaluated considering the 2 approaches of multifocal pain.

#### Step 3: Nociceptive Pain Cannot Be Entirely Responsible for the Pain

To date, there exist no grading systems or clearly defined criteria to decide whether nociceptive pain is fully responsible for the pain. Medical imaging, a thorough patient interview and physical examination could be used to decide whether nociceptive pain is present, but still cannot rule out the presence of concurrent nociplastic pain (mixed pain mechanism). The interpretation of this interview and examination still depends of the clinical subjective judgment of an investigator.^18^ This study measured KOA grade with x-ray or magnetic resonance (if no x-ray was available) images from the medical record before TKA surgery.^41,42^ However, as confirmed KOA on medical imaging alone is insufficient to judge whether nociceptive pain if fully responsible for the pain, every participant who fulfilled step 2 was transferred to step 4.

#### Step 4: Neuropathic Pain Cannot Be Entirely Responsible for the Pain

The proposed grading system for neuropathic pain was used to judge this criteria.^43^ However, patients needed to have a history of relevant neurological lesion or disease to transfer to the next step to examine the presence of neuropathic pain. Because experiencing neuropathic-like pain (which was verbally checked if at least one of neuropathic pain symptoms according to the patient interview part of Douleur Neuropathique-4 [DN-4]^44^ was present) was an exclusion criteria of the current study, it was unlikely that neuropathic-like pain was present in the current KOA sample. Therefore, all patients with KOA fulfilling step 3 were automatically transferred to step 5.

#### Step 5: Evoked Pain Hypersensitivity Phenomena

The IASP grading system defined the presence of evoked pain hypersensitivity if this could be elicited clinically in the region of pain by any one of the following: (1) static mechanical allodynia, (2) dynamic mechanical allodynia, (3) painful after-sensations, or (4) heat or cold allodynia.^18^ Therefore, the following methods were used in the current study:

1. Static mechanical allodynia was measured with pressure pain thresholds (PPTs) that were taken at the medial and lateral joint-spaces of the knee with a hand-held pressure algometer (Wagner FDX 25 Force Gage). The participant was lying supine, whereas the probe (1 cm^2^) was placed perpendicular to the test surface. Pressure was increased with a speed of 9.8 N/s until the subject reported a first feeling of pain/discomfort (1 of 10 on numeric pain rating scale) felt at the stimulus location. This was repeated after 30 seconds, and the average of both measurements was taken. Measuring PPT is found to be reliable and valid.^45^
2. No measurement for dynamic mechanical allodynia was available in the dataset. However, also a TS measurement was performed in the region of pain (the knee awaiting TKA). Therefore, we decided to add this variable to the judgment of step 5. Thirty pinpricks were given at the skin overlying the medial tibiofemoral joint line of the affected knee at a pace of 1 pinprick/second with a Von Frey monofilament of 60 g. Together with the first and the last stimulus, the subject was instructed to give a pain score felt at the stimulus location on a numeric rating scale (NRS) ranging from 0 to 10, where 0 indicated “no pain” and 10 indicated “unbearable pain.” The differences of the NRS scores were calculated and used for analysis. This method is found to be reliable.^46^
3. Painful after-sensations: after ending the 30 pinpricks with the Von Frey monofilament, a pause of 15 seconds followed, and after this pause, the patient had to score their pain felt at the stimulus location again on a NRS (without any stimulus given at that moment). This is also found to be reliable.^46^
4. Heat/cold allodynia: thermal rollers (Rolltemp II Somedic Senselab) having a temperature of 25°C (cold stimulus) and 40°C (hot stimulus) were used. The rollers were passed 10 seconds at the skin overlying the medial and lateral tibiofemoral joint line of the affected knee. After these 10 seconds, patients needed to score their sensation of pain again felt at the stimulus location on an NRS as described earlier. Thermal rollers have been used in previous research to test thermal allodynia in this population^25^ as 25°C and 40°C do not normally activate nociceptors.^47^ This method is also recommended to test abnormalities in thermal sensation.^48^

For applying this step, normative data (n=38) from another ongoing project aiming to establish reference values for quantitative sensory testing in healthy people were used. This project was approved by the Ethical committee of the University Hospital of Antwerp (BE3002021000016). Eligibility criteria for these pain-free people are also presented in Table 1. The exact same measurements as provided in the population with KOA were also applied in these pain-free participants at the Sensoric Functioning Lab at the University of Antwerp. For each patient with KOA, every value was compared with the mean and standard deviation of the pain-free population based on z-scores. If a z-score exceeded a value of 1.96, the value was considered indicative of evoked hypersensitivity,^49,50^ meaning that the participant fulfilled step 5 and was categorized as having at least “possible” nociplastic pain.

#### Step 6: History of Pain Hypersensitivity and Comorbidities

The IASP grading system defined the part “a history of pain hypersensitivity” as fulfilled when participants mentioned any of the following during a patient interview with the executive researchers (Belgium) or nurses (the Netherlands): sensitivity to (1) touch, (2) pressure, (3) movement, or (4) cold or heat.^18^ The part “presence of comorbidities” was defined as fulfilled when the participants presented with any one of the following: (1) increased sensitivity to sound and/or light and/or odors; (2) sleep disturbance with frequent nocturnal awakenings; (3) fatigue; or (4) cognitive problems such as difficulty to focus, attention, memory disturbances, etc.

In the current study, we had the opportunity to carry out an extensive quantitative sensory testing instead of a patient interview (which was included in the previous step). As such, we only considered the “presence of comorbidities” part for the fulfillment of step 6. As no validated methods or cutoff scores are defined yet, we followed the approach of Nijs et al^30,51^ and Foubert et al^52^ to assess and interpret this step by using the individual questions of the central sensitization index (CSI) part A. This questionnaire, in which every item is scored from 0 (never) to 4 (always), is found to be reliable.^53^ The CSI questions suggested to be related to the proposed comorbidities^30,51^ can be found in Table 2. Foubert et al^52^ defined the following cutoff criteria to objectively fulfill this criterium of “probable” nociplastic pain: a score of ≥3 (often or always present) for ≥2 of the selected CSI questions (Table 3).

**TABLE 2:** Extra Biopsychosocial Variables Used to Compare No and “Probable” Nociplastic Pain Groups (Apart From Variables Used in the IASP Grading System)

Biopsychosocial variable group	Variable	Measurement
Demographic variables	Age	Birth date until first physical measurement
	Sex	Man or woman
Metabolic and inflammatory variables	BMI	Weight/(length in cm)2
	HbA1c value	A1CNow+ system (PTS Diagnostics, China) and a fingerstick
	Fat and lean mass	Bioelectrical Impedance Analysis (Bodystat Quadscan 4000)
	C-reactive protein	Blood sample
Pain-related variables	Pain intensity	KOOS subscale pain and NRS pain in rest
	Pain symptoms	KSSS subscale symptoms
	PPT m. Tibialis anterior, forehead and m. ECRL	Hand-held pressure algometer (Wagner FDX 25 Force Gage)
	Temporal summation m. ECRL	Von Frey Monofilament 60 g
	Thermal allodynia at m. ECRL	Thermal rollers (Rolltemp II)
	CPM	Q-Sense CPM *(Medoc, USA)*
Functional variables	Isometric strength of m. Quadriceps and m. Hamstrings of the affected leg	MicroFET 2 hand-held dynamometer (ProCare, Groningen)
	Proprioception of the affected leg	Plurimeter (Dr. Rippstein, Switzerland)
	Functionality (symptoms and functional ability of performing activities)	KOOS subscale symptoms, 30-s timed chair stand test, KSSS functional score
Psychological variables	Pain catastrophizing	PCS total score, subscale rumination, magnification and helplessness
	Depression	HADS subscale depression
	Anxiety	HADS subscale anxiety
	Expectations about the surgery	KSSS subscale expectations
	Satisfaction about their current pain	KSSS subscale satisfaction
	Illness perceptions	IPQR subscale identity, consequences, timeline, personal control, treatment control, illness coherence, emotional representations
Structural variables	Grade of OA	RX or MRI
Social variables	Work Educational level Marital status	Demographic questionnaire scored on different levels

BMI indicates body mass index; CPM, conditioned pain modulation; ECRL, extensor carpi radialis longus; HADS, Hospital Anxiety and Depression Scale; HbA1c, glycated hemoglobin; IPQR, Illness Perceptions Questionnaire Tevised; KOOS, Knee Injury and Osteoarthritis Outcome Score; KSSS, Knee Society Scoring System; m, musculus; MRI, magnetic resonance imaging; NRS, numeric rating scale; OA, osteoarthritis; PCS, Pain Catastrophizing Scale; PPT, pressure pain threshold; RX, radiography; s, seconds.

**TABLE 3:** Additional Information for the Results of Steps 5 and 6 of the IASP Grading System

Additional information for step 5				
Approach 1: 4 pain locations	KOA (n=73), mean ± SD	Healthy (n=38), mean ± SD	Z-score <−1.96* or >1.96**, N (%)	Z-score <−1.96* or >1.96** on ≥ 1 QST item, N (%)
PPT medial knee (N)*	36.45 ± 20.90	64.87 ± 33.10	0 (0)	50 (68.49)
PPT lateral knee (N)*	40.69 ± 25.82	68.43 ± 35.23	0 (0)	
TS** (Diff in NRS)	1.58 ± 2.25	0.30 ± 0.55	34 (46.58)	
TS after sensation (NRS)**	0.81 ± 1.55	0.08 ± 0.22	20 (27.40)	
HPA medial knee (NRS)**	1.10 ± 1.80	0.16 ± 0.40	27 (36.99)	
HPA lateral knee (NRS)**	0.63 ± 1.56	0 ± 0	16 (21.92)	
CPA medial knee (NRS)**	0.58 ± 1.09	0.01 ± 0.08	19 (26.03)	
CPA lateral knee (NRS)**	0.49 ± 1.17	0 ± 0	15 (20.55)	
Approach 2: 3 pain locations	KOA (n = 120)	Healthy (n = 38)		
PPT medial knee (N)*	40.01 ± 22.15	64.87 ± 33.10	0 (0)	82 (68.33)
PPT lateral knee (N)*	44.38 ± 25.69	68.43 ± 35.23	0 (0)	
TS** (Diff in NRS)	1.32 ± 2.07	0.30 ± 0.55	47 (39.17)	
TS After sensation (NRS)**	0.53 ± 1.28	0.08 ± 0.22	24 (20)	
HPA medial knee (NRS)**	0.92 ± 1.57	0.16 ± 0.40	42 (35)	
HPA lateral knee (NRS)**	0.42 ± 1.25	0 ± 0	21 (17.50)	
CPA medial knee (NRS)**	0.42 ± 0.96	0.01 ± 0.08	23 (19.17)	
CPA lateral knee (NRS)**	0.33 ± 0.94	0 ± 0	19 (15)	
Additional information for step 6				
Approach 1: 4 pain locations	KOA (n = 50)			
	CSI item		N (%) of individuals with a score of ≥ 3	Amount of individuals that scored ≥3 on ≥2 CSI items
Sound/light/odors	Item 7: I am sensitive to bright lights.		7 (14)	30 (60)
	Item 20: Certain smells, such as perfumes, make me feel dizzy and nauseated.		2 (4)	
Sleep disturbance	Item 1: I feel tired and unrefreshed when I wake up from sleeping.		23 (46)	
	Item 12: I do not sleep well.		21 (42)	
	Item 22: My legs feel uncomfortable and restless when I am trying to go to sleep at night.		16 (32)	
Fatigue	Item 8: I get tired very easily when I am physically active.		26 (52)	
	Item 17: I have low energy.		14 (28)	
Cognitive problems	Item 13: I have difficulty concentrating.		8 (16)	
	Item 23: I have difficulty remembering things.		8 (16)	
Approach 2: 3 pain locations	KOA (n = 82)			
Sound/light/odors	Item 7: I am sensitive to bright lights.		14 (17.07)	46 (54.12)
	Item 20: Certain smells, such as perfumes, make me feel dizzy and nauseated.		6 (7.32)	
Sleep disturbance	Item 1: I feel tired and unrefreshed when I wake up from sleeping.		35 (42.68)	
	Item 12: I do not sleep well.		31 (37.80)	
	Item 22: My legs feel uncomfortable and restless when I am trying to go to sleep at night.		21 (25.61)	
Fatigue	Item 8: I get tired very easily when I am physically active.		36 (43.90)	
	Item 17: I have low energy.		18 (21.95)	
Cognitive problems	Item 13: I have difficulty concentrating.		13 (15.85)	
	Item 23: I have difficulty remembering things.		15 (18.29)	

* Z-score ≤ −1.95.

** Z-score > 1.96.

AS indicates after sensation; BMI, body mass index; CPA, cold pain allodynia; Diff, difference; HPA, heat pain allodynia; KOA, knee osteoarthritis; N, Newton; NRS, numeric pain rating scale 0-10; PPT, pressure pain threshold; QST, quantitative sensory testing; TS, temporal summation.

### AIM 2: Comparing Biopsychosocial Variables Among Participants With “Probable” and “Possible or No” Nociplastic Pain

Apart from comparing the above-mentioned variables, also differences in demographic, metabolic, functional, psychological, structural, social, and other pain-related variables measured at baseline in the longitudinal study (see Table 2) were evaluated comparing patients with “probable” versus “no or possible” nociplastic pain to get an extensive overview of all biopsychosocial characteristics. Details about the measurements and their clinimetric properties used to assess these biopsychosocial variables can be found in the Supplemental Material, Supplemental Digital Content 1, http://links.lww.com/CJP/B132.

### AIM 3: The Response to TKA 1-year Postoperative Compared Between Groups

The KOOS subscale pain at 1-year post-TKA was used to compare the response to TKA treatment (pain intensity score of operated knee) between the different identified “probable” and “possible or no” nociplastic pain groups.

### Statistical Analyses

All statistical analyses were performed using the IBM Statistical Package for Social Sciences Version 29 (SPSS, IBM Corporation), and R software (version 4.2.3) for multiple imputation. First, univariate outliers were checked with boxplots (less than quartile 1 to 1.5* interquartile range or more than quartile 3 to 1.5* interquartile range), if present these were checked in the digital database and on the measurement form and only deleted if unreasonable (not in between the expected range). Second, participants with missing data in one of the variables used in the grading system were deleted (as the participant could not be run through the whole grading system) (AIM 1).

Focusing on AIMs 2 and 3, missing data were accounted using multiple imputations (n=10 imputed datasets),^54^ except if more than 40% of data was missing.^55^ To compare all biopsychosocial variables between the 2 groups in each pain-location approach (3 or 4 pain locations), linear-mixed model analyses were used (multinomial logistic regression for categorical variables of more than 2 categories). Group (probable nociplastic pain vs. possible or no nociplastic pain), age and sex (covariates—except if age and sex were the independent variables themselves) were used as fixed effects (part 2). Sex and age were used as independent factors but also added as covariates for the other independent factors because we know sex and age can influence quantitative sensory testing,^56,57^ psychological and physical factors.^58–60^ In addition, the difference in TKA treatment between the groups was examined with linear-mixed model analyses of which group (3 or 4 pain locations), age, sex, and KOOS subscale pain preoperative score (covariates) were used as fixed effects (part 3). Normality of the residuals and homogeneity of variance were checked. Based on the 10 imputed datasets, 10 different *P* values were generated for each comparison per variable and their median value was reported.^61^ A Benjamini-Hochberg correction was applied to correct for multiple testing and the significance level was therefore set to *P* <0.017.^62^ Data are presented as estimated mean and 95% confidence interval (95% CI) for continuous variables and as frequency and percentage for categorical variables.

Finally, a sensitivity analysis was performed to strengthen our group separation choices by separating subjects with “possible” nociplastic pain from the “no” nociplastic pain group, and comparing the 3 groups. Statistical analyses were run again on all variables; however, results will not be discussed in detail as this is beyond the scope of this article. Again, a Benjamini-Hochberg correction was applied to correct for multiple testing, and the significance level was therefore set to *P* <0.019 (4 pain locations) and *P* <0.017 (3 pain locations).^62^

## RESULTS

### AIM 1: Result of IASP Grading System in Patients With KOA

Preoperative data of 223 patients with KOA were available to apply to the grading system. Of the patients with KOA, 11 had missing data for the KOOS subscale pain, 16 for the body chart, and 1 for the CSI. This resulted in 197 included participants having full data necessary to run through the grading system (some had missing data on multiple variables). This sample had a mean age of 65.4 years ± 7.7, and consisted of 95 women (48%).

#### The Classification of Nociplastic Pain

The number of KOA patients fulfilling each step of the grading system is presented in Figure 1.

#### Step 1: Chronic Pain (>3 Months)

Apart from 2 (1%) patients, all participants experienced significant pain.^34^ Therefore, sufficient arguments were available to transfer these 195 (99%) patients to the next step in the decision tree.

#### Step 2: Multifocal Rather Than Discrete Pain

1. Approach 1 (knee + 3 additional pain locations): the pain of 73 (37.4%) participants was defined as widespread/regional, and that of 122 (62.6%) participants was discrete.
2. Approach 2 (knee + 2 additional pain locations): the pain of 120 (61.5%) participants was defined as regional, and that of 75 (38.5%) participants was discrete.

#### Step 3: Nociceptive Pain Cannot Be Entirely Responsible for the Pain

As mentioned in the Materials and Methods section, although all participants had confirmed KOA on medical imaging in combination with regional, widespread, or multifocal pain rather than discrete pain (discrete pain filtered out in previous step) does not exclude the presence of nociplastic pain.^18^ Therefore, all participants fulfilling step 2 (73 [37.6% of 194] and 120 [61.9% of 194], respectively) were automatically transferred to step 4.

#### Step 4: Neuropathic Pain Cannot Be Entirely Responsible for the Pain

As provided in the Materials and Methods section, none of the participants had neuropathic-like pain symptoms according to the DN-4 patient interview because this was an exclusion criterion for current study. As both a neuroanatomically plausible location and neuropathic-like pain symptoms need to be present to examine if neuropathic pain is the definite pain mechanism,^43^ none of the participants fulfilled this step. Therefore, all participants fulfilling step 2 and 3 (73 [37.6% of 194] and 120 [61.9% of 194], respectively) were also automatically transferred to the next step.

#### Step 5: Evoked Pain Hypersensitivity Phenomena

Table 3 provides an overview of the results and z-scores used for this criterion.

1. Approach 1 (knee + 3 additional pain locations): 50 (68.5% of 73) participants had evoked pain hypersensitivity and as such fulfilled step 5, and 23 (31.5% of 73) experienced no hypersensitivity.
2. Approach 2 (knee + 2 additional pain locations): 82 (68.3% of 120) participants had evoked pain hypersensitivity and as such fulfilled step 5, and 38 (31.7% of 120) experienced no hypersensitivity.

#### Step 6: History of Pain Hypersensitivity and Comorbidities

1. Approach 1 (knee + 3 additional pain locations): 30 (60.0% of 50) participants had at least 2 comorbidities and were therefore categorized as having “probable” nociplastic pain. The other 20 (40.0% of 50) were categorized as having “possible” nociplastic pain.
2. Approach 2 (knee + 2 additional pain locations): 46 (54.1% of 82) participants had at least 2 comorbidities and were therefore categorized as having “probable” nociplastic pain. The other 36 (45.9% of 82) were categorized as having “possible” nociplastic pain.

### PART 2: Comparing Biopsychosocial Variables Among Participants With “Probable” and “Possible or no” Nociplastic Pain

#### Data Preprocessing

Every variable used to assess differences between groups had no (n=24 variables) or <4% (n=21 variables) missing data, except for 6 variables: PPT forehead had 23 (11.86%) missing values (because this variable was added later in the study protocol), conditioned pain modulation (CPM) 15 (7.62%) (because of device deficits or some participants reporting no pain during the test stimulus), and glycated hemoglobin (HbA1c) 16 (8.12%) (because of device deficits). Fat and lean mass and C-reactive protein had more than 40% missing values and were as such not added for multiple imputation and analysis.^55^ Baseline values and amount of missing values for the KOA patients can be found in the Supplemental Material, Supplemental Digital Content 1, http://links.lww.com/CJP/B132.

### Differences Between Groups

Details about group differences can be found in Tables 4 and 5.

**TABLE 4:** Differences Between Knee Osteoarthritis Participants Without and With “Probable” No Nociplastic Pain (Continuous Variables) at Baseline and 1-year Postoperative

	4 Pain locations			3 Pain locations		
Variable	Estimated mean (95%CI)			Estimated mean (95% CI)		
Continuous variables	Probable nociplastic pain (n=30)	Possible + no nociplastic pain (n=167)	*P*	Probable nociplastic pain (n=46)	Possible + no nociplastic pain (n= 151)	*P*
Demographic variable						
Age	61.83 (59.14-64.53)	65.98 (64.84-67.13)	0.006*	62.41 (60.24-64.58)	66.25 (65.05-67.44)	0.003*
Metabolic and inflammatory variables						
BMI (kg/m^2^)	29.88 (27.94-31.83)	29.99 (29.18-30.80)	0.919	29.90 (28.32-31.47)	30.00 (29.15-30.86)	0.911
Hba1c value (%)	5.65 (5.41-5.89)	5.57 (5.48-5.67)	0.577	5.67 (5.47-5.84)	5.56 (5.45-5.66)	0.361
Pain-related variables						
Body chart (N)	6.41 (5.72-7.09)	2.95 (2.67-3.24)	<0.001*	5.19 (4.59-5.79)	2.96 (2.63-3.28)	<0.001*
NRS pain in rest (0-10)	4.86 (3.88-5.84)	4.57 (4.16-4.98)	0.602	5.07 (4.28-5.86)	4.47 (4.05-4.90)	0.199
KOOS subscale pain (0-100)	38.09 (32.52-43.56)	44.88 (42.60-47.16)	0.027	39.12 (34.71-43.53)	45.29 (42.87-47.70)	0.019
PPT m. Tibialis anterior (Ne)	42.73 (35.08-50.39)	50.97 (47.79-54.15)	0.055	46.27 (40.05-52.49)	5.75 (47.38-54.13)	0.223
PPT MK joint line (Ne)	31.03 (23.28-38.78)	43.86 (40.64-47.08)	0.003*	33.52 (27.26-39.78)	44.46 (41.06-47.86)	0.003*
PPT LK joint line (Ne)	36.59 (28.17-45.01)	48.77 (45.27-52.27)	0.010*	41.11 (34.25-47.96)	48.68 (44.95-52.40)	0.063
PPT m. ECRL (Ne)	30.79 (25.19-36.39)	37.97 (35.54-40.30)	0.023	32.77 (28.23-37.31)	38.12 (35.66-40.59)	0.047
PPT forehead (Ne)	25.07 (20.48-29.65)	31.02 (29.02-33.02)	0.020	27.79 (24.01-31.57)	30.81 (28.72-32.91)	0.152
TS MK joint line (Diff in NRS)	2.21 (1.51-2.91)	1.10 (0.81-1.38)	0.005*	1.76 (1.19-2.33)	1.12 (0.81-1.43)	0.060
After sensation medial knee (0-10)	1.13 (0.72-1.53)	0.31 (0.14-0.48)	<0.001*	0.73 (0.40-1.07)	0.34 (0.16-0.52)	0.049
TS medial wrist (Diff in NRS)	1.82 (1.23-2.41)	0.93 (0.68-1.17)	0.007*	1.62 (1.15-2.10)	0.89 (0.63-1.45)	0.010*
After sensation medial wrist (0-10)	0.26 (0.04-0.49)	0.15 (0.06-0.25)	0.391	0.26 (0.04-0.49)	0.15 (0.06-0.25)	0.867
Cold allodynia MK joint line (0-10)	1.04 (0.69-1.38)	0.25 (0.10-0.39)	<0.001*	0.78 (0.49-1.06)	0.24 (0.09-0.40)	0.001*
Heat allodynia MK joint line (0-10)	1.85 (1.31-2.40)	0.71 (0.48-0.93)	<0.001*	1.51 (1.07-1.96)	0.69 (0.45-0.93)	0.002*
Cold allodynia LK joint line (0-10)	0.95 (0.61-1.30)	0.19 (0.04-0.33)	<0.001*	0.61 (0.32-0.89)	0.21 (0.05-0.37)	0.018
Heat allodynia LK joint line (0-10)	1.21 (0.80-1.62)	0.27 (0.10-0.45)	<0.001*	0.78 (0.44-1.12)	0.31 (0.12-0.50)	0.019
Cold allodynia m. ECRL (0-10)	0.51 (0.22-0.81)	0.15 (0.03-0.28)	0.029	0.35 (0.11-0.59)	0.17 (0.04-0.30)	0.182
Heat allodynia m. ECRL (0-10)	1.08 (0.65-1.51)	0.39 (0.21-0.57)	0.004*	0.84 (0.50-1.19)	0.39 (0.20-0.58)	0.027
CPM relative score (%)	7.24 (-17.29-31.77)	16.41 (5.75-27.07)	0.507	4.19 (-15.63-24.02)	18.33 (7.11-29.55)	0.207
CSI (0-100)	40.29 (36.12-44.47)	26.33 (24.56-28.07)	<0.001*	38.81 (35.53-42.10)	25.30 (32.50-27.08)	<0.001*
Functional variables						
Strength m. Quadriceps (kgf)	23.57 (19.54-27.50)	27.94 (26.21-29.57)	0.047	22.28 (19.18-25.38)	28.81 (27.12-30.50)	<0.001*
Strength m. Hamstrings (kgf)	10.00 (8.04-11.96)	12.19 (11.37-13.00)	0.048	10.48 (8.90-12.07)	12.27 (11.41-13.13)	0.058
Proprioception (degrees)	4.28 (3.51-5.06)	4.49 (4.18-4.81)	0.558	4.43 (3.90-5.15)	4.44 (4.10-4.78)	0.872
30-s chair stand test (N)	9.87 (8.39-11.35)	10.91 (10.29-11.53)	0.210	9.46 (8.27-10.65)	11.14 (10.50-11.79)	0.017
KSSS symptoms (0-20)	8.12 (6.46-9.79)	8.50 (7.81-9.20)	0.685	8.07 (6.73-9.42)	8.56 (7.83-9.29)	0.542
KSSS functional score (0-100)	37.60 (32.18-43.01)	44.01 (41.76-46.27)	0.035	37.60 (32.18-43.01)	44.01 (41.76-46.27)	0.035
KOOS subscale symptoms (0-100)	9.87 (8.61-11.13)	10.31 (9.79-10.84)	0.534	9.99 (8.98-11.01)	10.32 (9.77-10.88)	0.578
Psychological variables						
IPQR identitiy score (0-14)	2.21 (1.69-2.73)	2.11 (1.90-2.33)	0.727	2.49 (2.08-2.91)	2.01 (1.79-2.24)	0.050
IPQR timeline (6-30)	19.02 (17.08-20.97)	17.65 (16.84-18.46)	0.208	19.46 (17.90-21.02)	17.37 (16.52-18.22)	0.024
IPQR consequences (6-30)	19.33 (17.80-20.87)	19.46 (18.83-20.10)	0.879	20.63 (19.41-21.86)	19.08 (18.41-19.74)	0.032
IPQR personal control (6-30)	19.81 (18.33-21.28)	19.67 (19.06-20.28)	0.872	19.62 (18.43-20.80)	19.72 (19.07-20.36)	0.886
IPQR treatment control (5-25)	17.85 (16.71-18.98)	18.21 (17.74-18.68)	0.568	17.67 (16.76-18.58)	18.30 (17.81-18.80)	0.240
IPQR illness cohorence (5-25)	19.31 (18.53-20.09)	18.63 (18.30-18.95)	0.117	19.21 (18.58-19.84)	18.59 (18.24-18.93)	0.096
IPQR timeline cyclical (4-20)	11.90 (10.49-13.32)	11.97 (11.38-12.56)	0.937	11.28 (10.14-12.42)	12.17 (11.55-12.79)	0.188
IPQR emotional representations (6-30)	17.63 (15.98-19.28)	15.49 (14.80-16.17)	0.021	17.58 (16.26-18.90)	15.28 (15.56-15.99)	0.003*
PCS rumination (0-16)	7.43 (6.03-8.83)	6.05 (5.47-6.63)	0.078	7.30 (6.17-8.42)	5.94 (5.33-5.56)	0.042
PCS magnification (0-12)	3.67 (2.76-4.61)	2.55 (2.17-2.93)	0.028	3.86 (3.12-4.59)	2.38 (1.98-2.77)	<0.001*
PCS helplesness (0-24)	9.10 (7.26-10.93)	7.05 (6.29-7.82)	0.047	9.52 (80.6-1098)	6.70 (5.90-7.49)	0.001*
PCS total score (0-52)	20.21 (16.44-23.98)	15.65 (14.08-17.22)	0.031	20.67 (17.67-23.68)	15.02 (13.38-16.65)	0.002*
HADS fear (0-21)	7.10 (5.70-8.49)	5.02 (4.44-5.60)	0.008*	6.67 (5.54-7.79)	4.93 (4.32-5.54)	0.010*
HADS depression (0-21)	6.88 (5.73-8.02)	4.74 (4.27-5.22)	0.001*	6.55 (5.63-7.47)	4.42 (4.11-5.12)	<0.001*
KSSS satisfaction (0-40)	12.98 (10.35-15.60)	15.80 (14.70-16.89)	0.056	13.68 (11.55-15.80)	15.88 (14.72-17.04)	0.080
KSSS expectations (30-15)	13.48 (12.89-14.07)	14.04 (13.49-14.28)	0.090	13.60 (13.12-14.07)	14.06 13.80-14.32)	0.101
One-year postoperative outcome variable						
KOOS subscale pain	60.23 (50.08-70.37)	74.27 (69.94-78.61)	0.005*	62.83 (55.14-70.52)	74.97 (70.41-79.53)	0.004*

* Blue is significant difference (*P*<0.017). All variables are adjusted for sex and age (except age itself).

BMI indicates body mass index; CPM, conditioned pain modulation; CSI, Central Sensitization Inventory; Diff, difference; ECRL, extensor carpi radialis longus; HADS, hospitality anxiety and depression scale; Hb1ac, glycated hemoglobin; IPQR, illness perceptions questionnaire revised; kg/m2, kilograms/squared meter; kgf, kilograms force; KOOS, Knee Injury and Osteoarthritis Outcome Scale; KSSS, Knee Society Scoring System; LK, lateral knee; m, musculus; MK, medial knee; Ne, Newton; NRS, numeric rating scale; PCS, Pain Catastrophizing Scale; PPT, pressure pain threshold; TS, temporal summation.

**TABLE 5:** Differences Between Knee Osteoarthritis Participants Without and With “Probable” No Nociplastic Pain (Categorical Variables)

	4 Pain locations			3 Pain locations		
Categorical variables	N (%)			N (%)		
Variable	Probable nociplastic pain (n=30)	Possible + no nociplastic pain (n=167)	*P*	Probable nociplastic pain (n=46)	Possible + no nociplastic pain (n=151)	*P*
Demographic variable						
Sex						
Man	9 (30.00)	93 (55.69)	0.009*	16 (34.78)	86 (56.95)	0.008*
Woman	21 (70.00)	74 (44.31)		30 (65.22)	65 (43.05)	
Structural variable						
Grade of KOA						
K&L 1	1 (3.33)	2 (1.20)	0.116	3 (6.52)	0 (0.00)	0.063
K&L 2	10 (33.33)	32 (19.16)		14 (30.43)	28 (18.54)	
K&L 3	10 (33.33)	61 (36.53)		13 (28.26)	57 (37.75)	
K&L 4	9 (30.00)	72 (43.11)		16 (34.78)	66 (43.71)	
Social variables						
Education						
No degree	3 (10.00)	8 (4.97)	0.994	3 (6.52)	8 (5.52)	0.603
Primary school	1 (3.33)	10 (6.21)		2 (4.35)	9 (6.21)	
Technical secondary school	5 (16.67)	41 (25.47)		13 (28.26)	33 (22.76)	
Higher secondary school	3 (10.00)	21 (13.04)		5 (10.87)	19 (13.10)	
High school	9 (30.00)	39 (24.22)		10 (21.74)	38 (26.21)	
University	3 (10.00)	13 (8.07)		3 (6.52)	13 (8.97)	
Other	6 (20.00)	35 (21.74)		10 (21.74)	31 (21.38)	
Work						
Pension	9 (30.00)	95 (59.01)	0.567	16 (34.78)	88 (60.69)	0.463
Self-employed	5 (16.67)	9 (5.59)		6 (13.04)	8 (5.52)	
White-collar worker	6 (20.00)	20 (12.42)		9 (19.57)	17 (11.72)	
Laborer	4 (13.33)	21 (13.04)		6 (13.04)	19 (13.10)	
Unemployed	0 (0.00)	2 (1.24)		0 (0.00)	2 (1.38)	
Other	6 (20.00)	19 (11.80)		9 (19.57)	16 (11.03)	
Marital status						
Married	20 (66.67)	121 (75.16)	0.841	32 (69.57)	109 (75.17)	0.817
Divorced	3 (10.00)	14 (8.70)		4 (8.70)	13 (8.97)	
Single	3 (10.00)	5 (3.11)		3 (6.52)	5 (3.45)	
Widow(er)	1 (3.33)	17 (10.56)		2 (4.35)	16 (11.03)	
Other	3 (10.00)	9 (5.59)		5 (10.87)	7 (4.83)	

* Blue is significant difference (*P*<0.017). All variables are adjusted for age and sex (except sex itself).

K&L indicates Kellgren and Lawrence scale.

#### Approach 1: Four Pain Locations

After running through the IASP grading system, 30 participants (15.23%) were classified as having “probable” nociplastic pain. The “probable” nociplastic pain group included more woman (*P*=0.010), and had a lower age (*P*=0.006), higher number of pain locations (*P*<0.001), lower PPT at medial (*P*=0.003) and later knee joint line of affected knee (*P*=0.010), higher thermal allodynia seconds at the skin overlying the medial and lateral tibiofemoral joint line of the affected knee (all *P*<0.001), higher TS (*P*=0.005) and after sensation (*P*<0.001) at the skin overlying the medial tibiofemoral joint line of the affected knee, higher TS at medial wrist (*P*=0.007), higher heat allodynia measured at m. extensor carpi radialis longus (*P*=0.004), higher CSI scores (*P*<0.001), and higher anxiety (*P*=0.008) and depression scores (*P*=0.001) compared with the “possible or no” nociplastic pain group. Other variables were nonsignificant (*P*>0.05). The sensitivity analysis revealed similar results, except that age and TS measured at the skin overlying the lateral knee were not significantly different anymore (*P*>0.019). Post hoc testing showed that differences were mostly present between the “probable” and the “no” nociplastic pain group (Supplemental Material, Supplemental Digital Content 1, http://links.lww.com/CJP/B132).

#### Approach 2: Three Pain Locations

Using this method, 46 participants (23.35%) were classified as having “probable” nociplastic pain. The “probable” group included more woman (*P*=0.007), and had a lower age (*P*=0.003), a higher number of pain location (*P*<0.001), lower PPT (0.003) and higher cold (*P*=0.001), and heat (*P*=0.002) allodynia at the skin overlying the medial tibiofemoral joint line of affected knee, higher TS at medial wrist (*P*=0.010), higher CSI score (*P*<0.001), higher scores of the Illness Perceptions Questionnaire Revised (IPQR) subscale emotional representations (*P*=0.003), the subscale magnification (*P*<0.001), helplessness (*P*=0.001) and total score (*P*=0.002) of the Pain Catastrophizing Scale (PCS), subscale anxiety (*P*=0.010) and depression (*P*<0.001) of the Hospital Anxiety and Depression Scale (HADS). [Author needs to provide a reference for the IPQR, PCS, HADS, and KSS,] and lower m. Quadriceps strength (*P*<0.001) compared with the “possible or no” nociplastic pain group. Other variables were not different between groups (*P*>0.05). The sensitivity analysis revealed similar results except for 5 variables: TS (*P*=0.010) and after-sensations (*P*=0.007) measured at the skin overlying the medial tibiofemoral joint line of the affected knee, and the functional score of the Knee Society Scoring System (KSSS) (*P*=0.010), which appeared to be significantly different and were worse in the “probable” nociplastic pain group. TS measured at the skin overlying the medial wrist and HADS fear were not significant anymore. Post hoc testing showed again that differences were mostly present between the “probable” and the “no” nociplastic pain group (Supplemental Material, Supplemental Digital Content 1, http://links.lww.com/CJP/B132).

### Part 3: The Response to TKA 1-year Postoperative Compared Between Groups

The KOOS subscale pain measured at 1-year post-TKA had missing data for 41 participants (20.8%) because participants had no time for the measurements or were unreachable (n=40) or were planned for revision surgery (n=1). Baseline values and number of missing values can also be found in the Supplemental Material, Supplemental Digital Content 1, http://links.lww.com/CJP/B132. The “probable” group had lower KOOS subscale pain scores (= more pain) at baseline compared with the “possible or no” nociplastic pain group (however, not significant after Benjamini-Hochberg correction) and was therefore used as a covariate in the analysis. For both pain locations approaches, the “probable” group had lower KOOS subscale pain scores (= more pain) compared with the “possible or no” nociplastic pain group (*P*=0.005 for approach 1 to 4 pain locations, *P*= 0.004 for approach 2 to 3 pain locations) 1-year post-TKA (Table 4). The sensitivity approach showed the same results (Supplemental Material, Supplemental Digital Content 1, http://links.lww.com/CJP/B132).

## DISCUSSION

The first aim was to apply the IASP grading system and identify nociplastic pain in patients with KOA. Two approaches were used to interpret regional pain: approach 1 included pain at the affected knee and 3 additional locations, whereas approach 2 only included 2 additional locations. Among 197 patients with KOA, 15.2% (approach 1) or 23.4% (approach 2) were categorized with “probable” nociplastic pain. More detailed criteria were proposed to interpret every step of the grading system, except for discrete/regional/multifocal/widespread pain, for which no recommendation for 3 or 4 pain locations could be given yet. The second aim was to compare biopsychosocial factors between participants with “possible or no” and “probable” nociplastic pain. In both approaches the “probable” group included more woman, had lower age, a higher number of pain locations, higher widespread TS, higher CSI scores, higher thermal allodynia measured at the skin overlying the medial knee joint line and more anxiety and depression compared with “possible or no” nociplastic pain group. In approach 1, the “probable” nociplastic pain group also exhibited characteristics such as lower local PPT and higher thermal allodynia measured at the skin overlying the lateral knee joint line, higher local TS and after sensation, and higher widespread heat allodynia. In approach 2, the “probable” nociplastic pain group also exhibited lower PPT, worse illness perceptions about emotional representations and m. Quadriceps strength, and higher magnification, helplessness and general pain catastrophizing. The third aim was to compare the response to TKA treatment between groups. The “probable” nociplastic pain group had more pain compared with the other group 1-year post-TKA. Sensitivity analyses revealed comparable results.

### Interpretation of Findings and Relation to Previous Research

This study found that the IASP grading system is feasible for identifying and characterizing patients with KOA with nociplastic pain. However, challenges emerged in executing its application. First, the use of terms such as “regional/multifocal/widespread” (which are not necessarily synonyms^63–65^), and information about the specific pain distribution area (which was defined as “rather cutaneous and regional, multifocal, or widespread in distribution [rather than discrete]”), lacked clarity.^18^ Although previous research has provided some (unvalidated) thresholds for defining widespread pain in patients with KOA,^37,38^ clear cutoff criteria for regional pain have not been established.^65,66^ To address this issue, 2 approaches of having knee pain along with additional locations were used. This additional approach expanded knee pain plus solely 1 supplementary location because a significant number of patients with KOA experience concurrent contralateral KOA pain, which made the inclusion of at least 2 extra pain locations imperative.^39,40^ However, body locations were only counted and as such no information about “more regional” or “more widespread” pain could be given. Therefore, no recommendation could be made to use 3 or 4 pain locations as adequate to judge this step. More research is needed as this two-fold presentation only highlights the need for clearer criteria to judge this step in the future. Second, clear guidelines or a grading system to study whether nociceptive pain is entirely responsible for the pain is currently lacking but necessary for implementing and interpreting the comprehensive patient interview and full physical examination of the patient. Therefore, we had not enough arguments to say that nociceptive pain was entirely responsible for the pain in the participants who reached step 2.^18^ Third, an interpretation for “evoked hypersensitivity” and “history of comorbidities” was introduced based on previous literature, but further validation is required.^30,49,50,52^ In terms of comorbidities, a different cutoff as specified in the IASP grading system was chosen.^18^ Instead of relying solely on patient interviews, the CSI items were used because they covered all comorbidities outlined by the grading system. However, they are formulated with less stringency and are often mentioned as being common. For instance, “I feel tired and unrefreshed” or “getting tired very easily when physically active” are frequently reported among the KOA population given the age or physical condition but do not necessarily indicate the presence of “fatigue.”^67^ Moreover, the current study used a cutoff score of ≥3 (often or always present), but discussion remains present whether a score of 2 (sometimes present) may be sufficient to be classified as “having the comorbidity.” Finally, participants presenting with “possible” or “no” nociplastic pain were merged to 1 group because the presence of evoked pain hypersensitivity alone (“possible” group) does not automatically classify the pain as predominantly nociplastic pain.^68,69^ Thereupon, the aim of the current manuscript was to identify participants with a predominant nociplastic pain mechanism (and not with a “possible” nociplastic pain mechanism).

Previous research provided theoretical guidelines for the grading system in cancer^51^ and post-COVID contexts,^70^ but was based on solely theoretical considerations. No studies, except one preprint in patients with MSK disorders^50^ and 1 study in patients with hemophilia^52^ applied the IASP grading system to real patient datasets. Foubert et al^52^ found no differences regarding demographic, psychological, functional and quality of life between the “probable or possible” and “no” nociplastic pain group. A possible explanation could be the different pathology (hemophilia), but also their group comparison. Due to their small sample size, they decided to merge the “probable” with the “possible” nociplastic pain group, whereas the current study merged the “possible” with the “no” nociplastic pain group. The preprint that also tried to apply the grading system,^50^ categorized only 5% of their osteoarthritis sample as having “probable” nociplastic pain, but the small sample size (21 participants) and reliance solely on medical imaging to decide if the pain was predominantly nociceptive could explain the discrepancies with our findings. No analyses to study differences between pain mechanism groups and their response to treatment were performed. However, studies have been published comparing patients with KOA with a predominant neuropathic-like pain mechanism and a predominant nociceptive pain mechanism,^22–24^ or comparing patients with TKA with a predominant nociceptive, predominant pain sensitization, and mixed pattern.^25^ The proportions of patients with KOA with neuropathic-like pain (30% to 58% of participants)^22–24^ were higher compared with the “probable” nociplastic pain group in the current study (15% to 23% of participants), but more similar with the pain sensitization group (25%) in the latter study.^25^ This is plausible, as the studies comparing neuropathic-like pain with nociceptive pain also indicate that individuals with a nociplastic pain mechanism were part of this neuropathic-like pain group.^22–24^ Similar to our findings, KOA patients in the neuropathic-like pain^22–24^ or patients with TKA in the pain sensitization group^25^ experienced more disturbed somatosensory functioning,^23–25^ higher pain scores and number of pain locations,^22,25^ or more pain post-TKA.^23,24^ Similar to participants classified to our approach 2 (3 pain locations), participants in the study of Soni et al^24^ also experienced higher pain catastrophizing. The study of van Helvoort et al^22^ also found less radiographic damage in the participants with neuropathic-like pain, which could not be detected in our study. Caution is advised for interpreting these comparisons, as patients with KOA experiencing neuropathic-like pain were excluded from the current study.

In this KOA-focused study, 30 to 46 participants (15.22% to 23.35%) were categorized as “probable” nociplastic pain, which is a rather small sample size for our statistical analyses. However, our results are still of value in attempting to gain more insight into the characterization of patients with KOA with a predominant “probable” nociplastic pain mechanism according to the IASP grading system because of their consistency with the findings of a previous literature review and Delphi consensus expert study,^19,26^ and the larger sample sizes in each group compared with previous pain mechanism phenotype studies.^22–25^

Finally, our study showed that the “probable” nociplastic pain group had worse TKA outcome compared with the other group. It was indeed expected that TKA would not resolve all the pain reports, because in a predominant nociplastic pain mechanism, the pain is not (fully) related to tissue damage (KOA).^17^ Therefore, it is postulated that other treatment modalities focusing on a more comprehensive modern neuroscience approach are additionally required to further resolve the patient symptoms.^18,29^

### Strengths and Limitations of the Study

This study possesses several notable strengths. First, it is one of the first studies applying the IASP grading system to a real dataset going beyond mere theoretical descriptions,^51^ and the first one in patients with KOA specifically. Furthermore, it also presents differences in treatment outcome of which the characteristics of both subgroups can be valuable in clinical practice to inform shared decision making about perioperative treatment. Last, it addresses the crucial aspect of defining and providing suggestions for criteria and cutoffs for every step (except for discrete/regional/multifocal/widespread pain) based on reliable measurement methods as indicated by the creators of the IASP grading system.^18^ However, this paper also has some limitations. First, patients with KOA with neuropathic-like pain were excluded, and all patients were awaiting TKA, this makes that our sample is not representative for the general KOA population. Second, this was a secondary analysis of another longitudinal prospective study. This makes that an objective measure questioning pain duration, a comprehensive assessment for pathology at other painful sites, and a measurement for dynamic mechanical allodynia were not available in the current dataset. Nevertheless, given that KOA is a chronic disease, all participants were awaiting TKA, and the use of the proposed cutoff of pain or not,^34^ we argue that there is sufficient rationale to categorize the pain as ≥3 months. In addition, TS was used as alternative for dynamic mechanical allodynia, because this measurement was also performed in the region of pain. Third, the number of pain locations was only counted, without the presentation of a pain drawing (step 2). Therefore, no information about whether the pain location was regional or widespread could be provided. As such, further research is necessary to provide a recommendation for clinical practice. However, the grading system itself does not provide specific criteria for assessing this step, so presenting 2 approaches for a cutoff was a first suggestion. Fourth, 1 patient was planned for revision surgery, suggesting that this missing value was not missing at random regarding the KOOS subscale pain score 1-year post-TKA. However, all other missing values were missing at random. Finally, originally, the test stimulus in the CPM measurement had to be a temperature equal to a pain intensity of 4 of 10,(71) but only participants reporting 0 of 10 were excluded in the current analysis. Therefore, it is possible that the stimulus was not noxious enough to elicit a CPM effect in some participants.

### Implications for Further Research and Clinical Practice

Further research is warranted to validate and further refine the IASP grading system (especially to interpret discrete/regional/multifocal/widespread pain), replicate our interpretation with external validation, and investigate if the proposed IASP grading system is applicable to other patients with MSK using our more specific approach. In clinical practice, our approach holds potential value for clinicians in making informed decisions about the presence of nociplastic pain in KOA patients and shared decision making about the perioperative treatment. As such, clinicians can decide whether these patients require additional or alternative treatments such as cognitiv- behavioral therapy, pain neuroscience education, and exposure in vivo.^69^

## CONCLUSIONS

The current study proposed more refined criteria for the grading system of nociplastic pain (except for discrete/regional/multifocal/widespread pain) and found that a significant portion of participants, ranging from 15.22% to 23.35%, could be categorized as having “probable” nociplastic pain according to the IASP grading system. Irrelevant of which pain distribution approach was used, the ‘probable’ nociplastic pain included more woman, participants with a lower age, a higher preoperative number of pain locations, widespread TS, higher CSI scores, higher thermal allodynia measured at the skin overlying the medial knee joint line, anxiety and depression compared with the “possible or no” nociplastic pain group. In addition, participants in the “probable” nociplastic pain group experienced more pain 1-year after TKA compared with the other group. More research is necessary to validate and to propose suggestions to improve the grading system itself.

## Supplementary Material

SUPPLEMENTARY MATERIAL
