# Supplementary material for: Application of the IASP Grading System to Identify Underlying Pain Mechanisms in Patients With Knee Osteoarthritis: A Prospective Cohort Study
Source: Clin J Pain. 2024 Jul 17;40(10):563–77. doi: 10.1097/AJP.0000000000001234 (PMC11389887; doi:10.1097/AJP.0000000000001234)
Supplement: SUPPLEMENTARY MATERIAL [file ajp-40-563-s001.docx]

**Supplementary material**

**Supplementary table S1 + references of the table:**

| **Variable** | **Measurement method** | **-Measurement device**  **-Data type**  **-Scoring**  **-Reference to psychometric properties** |
| --- | --- | --- |
| **Demographic factors** | | |
| **Age** | Birth date until first physical measurement | -Demographic questionnaire  -Continuous variable  -/ |
| **Sex** | Man or woman | -Demographic questionnaire  -Nominal variable |
| **Structural factors** | | |
| **Grade of KOA** | -X-ray images in AP, profile and Rosenberg weight-bearing position (1).  -Retrospectively extracted from the participant’s record by the general practitioner of the participants or the participants themselves  -If one of the images was not available, scoring was based on the available image(s). If no X-ray image was available, MRI in coronal and sagittal position were extracted. If none of the X-ray or MRI images could be found, this variable was recorded as missing value. | -K&L scale (2) or MRI grading system (3)  -Ordinal variable  -5-point Likert scale: 0 (no KOA) to 4 (worst grade of KOA)  -K&L: Good reliability and validity in KOA (4)  MRI grading: Good reliability and responsiveness (5) |
| **Metabolic and inflammatory factors** | | |
| **BMI** | -Length: demographic questionnaire  -Weight: standing on an electronic scale at the moment of testing | -Formula: Weight/(length in cm)^2  -Continuous variable  -kg/cm^2  -N/A |
| **HbA1c** | -Sitting position  -Taking a blood sample by pricking into a fingertip | -A1CNow+ system (*PTS Diagnostics, China*) and a fingerstick (6)  -Continuous variable  -%  -Accurate measurement to detect diabetes (7) |
| **Fat mass** | -Supine lying position  -Skinfold electrodes on hand and foot connected to the device | -Bioelectrical Impedance Analysis (Bodystat Quadscan 4000)  -Continuous variable  -N/A  -Accurate measurement to measure body composition (8) |
| **Lean mass** |  |  |
| **C-reactive protein** | -Blood sample before surgery, retrospectively extracted from participant’s record by executive researchers | -Blood sample  -Continuous variable  -mg/L  -Reliable method (9) |
| **Functional variables** | | |
| **Strength m. Quadriceps** | -Sitting position with hip and knee in 90°, upper leg fully supported by the table, and arm crossed over their chest. Isometric strength measurement was assured by using a traction belt.  -Perform flexion (Hamstrings) or extension (Quadriceps) of the knee against the device  -3 times, highest value used for analysis | -MicroFET 2 hand-held dynamometer (*ProCare, Groningen*)  -Continuous variable  -Kgf  -Reliable and valid (10) |
| **Strength m. Hamstrings** |  |  |
| **Proprioception** | -Sitting position with hip and knee in 90°, upper leg fully supported by the table  -Repositioning error during a knee joint position sense test (20°, 45° and 70° flexed knee)  -Twice assessed, mean of 6 trials used for analysis | -Plurimeter *(Dr. Rippstein, Switzerland)*  -Continuous variable  -° of knee angle  -Reliable (11) |
| **Functional symptoms** | -Questionnaire: questions related to stiffness, noises and mobility of the knee | -KOOS subscale symptoms  -Continuous variable  -5-point Likert scale: 0 (no symptoms) to 4 (always symptoms) for question 1 to 5, 4 (always) to 1 (never) for question 6 and 7  -Valid and reliable (12) |
| **Physical function** (13,14) | -Questionnaire: asking questions related to different activities  -Sum of subscales ‘walking and standing’, ‘standard activities’, ‘advanced activities’ and ‘discretionary activities | -KSSS Functional Score  -Continuous variable  -Scored 0 (impossible to perform any activities) – 120 (possible to perform any activity)  -Valid and reliable (15) |
|  | -Sitting position with arms resting next to the body  -Standing up and again sitting down as much as possible without support in 30s | -30 CST  -Continuous variable  -Number of times to stand up  -Reliable (16) |
| **Pain-related variables** | | |
| **Pain intensity** | -Questionnaire: questions related to pain intensity and specific movements during previous months of the knee that would undergo surgery | -KOOS subscale pain  -Continuous variable  -5-point Likert scale: 0 (no pain) to 4 (unbearable pain)  -Valid and reliable (12) |
|  | -Scale to measure pain intensity in rest at one moment | -Numeric pain rating scale  -Continuous variable  -11-point Likert scale: 0 (no pain) to 10 (unbearable pain)  -Valid and reliable (17) |
| **Pain symptoms** | -3 scales related to pain during walking on ground, pain during walking on stairs and how ‘normal’ the knee feels | -KSSS Symptom Score  -Continuous variable  -Scored 0 (no pain) – 25 (worst pain)  -Valid and reliable (15) |
| **Sensitization associated symptoms** | -Questionnaire: questions related to self-reported central sensitization  -Total score was used for analysis | -Central Sensitization Inventory  -Continuous variable  -5-point Likert scale: 0 (no central sensitization present) to 4 (most central sensitization symptom present)  -Reliable (18) |
| **Number of pain locations** | To draw their pain on a body chart by crossing all body parts that were painful during the last week | -Pain drawings on body chart  -Continuous variable  -Number of body parts  -Valid and reliable (19) |
| **Mechanical PPT** (13,20,21) | -Supine lying position  -The probe (1cm2) was placed perpendicular to the test surface and pressure increased with a speed of 9.8 Newton/second until the subject reported a first feeling of pain/discomfort at the location of the stimulus (+/- 1/10 NRS).  -Repeated after 30s (2 trials), mean used for analysis  Location:  Local hyperalgesia:  - Medial joint-space dominant side  - Lateral joint-space dominant side  Widespread hyperalgesia:  - m. Tibialis Anterior dominant side  - m. ECRL of non-dominant side  - Forehead | -Hand-held pressure algometer *(Wagner FDX 25 Force Gage, USA)*  -Continuous variable  -Newton/second  -Reliable (22,23) |
| **Thermal allodynia** | -Supine lying position  -At the skin overlying the medial and lateral joint-space of the affected knee (local thermal allodynia) and m. ECRL of non-dominant side (widespread thermal allodynia)  -The executive researcher rolled the thermoroller for 10s over the skin and participant had to score their pain intensity felt at the location of the stimulus | -Thermal rollers (Rolltemp II) with a roller of 25°C and 40°C  -Continuous variable  -NRS: 0 (no pain) to 10 (unbearable pain)  -Recommended to test abnormal thermal sensation (24) |
| **Temporal summation** | -Supine lying position  -At the skin overlying the medial joint-space of the affected knee (local temporal summation) and the dorsal wrist of the affected side (widespread temporal summation)  -30 repeated pinpricks with pace of 1 pinprick/s  -Pain NRS score felt at the location of the stimulus given by subject on first and last pinprick the subject  -The differences of the NRS scores were calculated and used for analysis. | - Von Frey monofilament 60g  -Continuous variable  -NRS: 0 (no pain) to 10 (unbearable pain)  -Reliable (25,26) |
| **CPM** | -Sitting position, lower arms supported, heat thermodes around the participant’s wrist  -The device searched for a temperature equal to a pain intensity NRS score of 4/10 (until a maximum of 46°C). This identified temperature (or 46°C when the 4/10 on a NRS was not reached) was used as test stimulus. The participant had to score the test stimulus on a NRS 4 times. After a pause of 120 seconds, a conditioning stimulus (with a temperature of 0.5°C more than the test stimulus) was added for 65 seconds and 20 seconds after its initiation, the test stimulus was repeated. Again, the participants had to score their pain for 4 times, but only on the test site. If the NRS at 46°C and the mean of the NRS of test stimulus was equal to zero, the participant was excluded for analysis of this variable. -Percentage change ((absolute score/NRS score during test stimulus)*100) scores were used for analysis. | -Q-sense CPM (*Medoc, USA*)  -Continuous variable  -NRS: 0 (no pain) to 10 (unbearable pain)  -Reliability to better confirmed(26) |
| **Psychological variables** | | |
| Pain catastrophizing (13,14,21,27) | -Questionnaire: questions related to pain catastrophizing  -Three subdomains: magnification, rumination and helplessness  -Total score was used for the analysis | -Pain Catastrophizing Scale  -Continuous variable  -5-point Likert scale: 0 (not at all) to 4 (all the time)  -Valid and reliable (28,29) |
| Depression | -Questionnaire: questions related to depression and anxiety  -Two subscales : depression and anxiety  -Scores of two subscales were used for analysis | -Hospital Anxiety and Depression Scale  -Continuous variable  -4-point Likert scale: 0 to 3 (variable meaning per item)  -Valid and reliable (30) |
| Anxiety |  |  |
| Expectations | -Questionnaire: questions related to surgery result expectation  -Subscale ‘expectations’ was used for analysis | -Knee Society Scoring System Score  -Continuous variable  -6-point Likert scale: 0 (no expectation) to 5 (high positive expectations)  -Valid and reliable (15) |
| Satisfaction | -Questionnaire: questions related to satisfaction about knee complaint  -Subscale ‘satisfaction’ was used for analysis | -Knee Society Scoring System Score  -Continuous variable  -5 items scored from 0 (no expectation) to 8 (high positive expectations)  -Valid and reliable (15) |
| Consequences | -Questionnaire: questions related to consequences of KOA complaint | - Illness perception questionnaire: subscales  -Continuous variable  -6 items scored from 1 (strongly disagree) to 5 (strongly agree)  -Reliable, expect for subscale cohorence (31) |
| Timeline | -Questionnaire: questions related to timeline of KOA complaint |  |
| Personal control | -Questionnaire: questions related to personal control over the KOA disease |  |
| Treatment control | -Questionnaire: questions related to treatment control over the KOA treatment |  |
| Emotional representation | -Questionnaire: questions related to emotional representation |  |
| Illness cohorence | -Questionnaire: questions related to illness coherence |  |
|  |  |  |
| Identity | -Questionnaire: questions related to experienced symptom related (or not) to the disease | -Illness perception questionnaire: subcale identity  -Continuous variable  -9 symptoms related to illness scored 0 (no) or 1 (yes)  -Reliable (31) |
| Causes of KOA | -Questionnaire: questions related to causes of KOA complaint | - Illness perception questionnaire: subcale causes  -Continuous variable  -Every question scored separately from 1 (strongly disagree) to 5 (strongly agree), no total score  -Reliable (31) |
| **Social variables** | | |
| Work | -Work level including pension, self-employed, white-collar worker, laborer, unemployed, or other | - Demographic questionnaire  -Nominal variable  -Scored from 1 to 6 |
| Education | -Educational level going from no degree, primary school degree, technical secondary school degree, higher secondary school degree, high school degree, university degree to other | - Demographic questionnaire  -Ordinal variable  -Scored from 1 to 7 |
| Marital status | -Marital status including married, divorced, single, widow(er) or other | - Demographic questionnaire  -Nominal variable  -Scored from 1 to 5 |
| Abbreviations: BPS = biopsychosocial, KOA = knee osteoarthritis, AP = anterior-posterior, MRI = magnetic resonance images, K&L scale = Kellgren and Lawrence scale, N/A = not applicable, BMI = body mass index, HbA1c = glycated hemoglobin (presence of diabetes type 2), KSSS = Knee Society Scoring System, s= second, 30CST = 30s timed chair stand test, KOOS = Knee Osteoarthritis Outcome and Index Score, ECRL = Extensor capri radialis longus, g = grams, NRS = numeric rating scale, h = hour, PPT = pressure pain threshold, CPM = conditioned pain modulation, kgf = kilogram force, mg/l = milligrams/liter, SPS = somatosensory processing signs | | |

1. Mortensen JF, Kappel A, Rasmussen LE, Østgaard SE, Odgaard A. The Rosenberg view and coronal stress radiographs give similar measurements of articular cartilage height in knees with osteoarthritis. Arch Orthop Trauma Surg. 2022 Sep;142(9):2349–60.

2. Felson DT, Niu J, Guermazi A, Sack B, Aliabadi P. Defining radiographic incidence and progression of knee osteoarthritis: suggested modifications of the Kellgren and Lawrence scale. Ann Rheum Dis. 2011 Nov;70(11):1884–6.

3. Park HJ, Kim SS, Lee SY, Park NH, Park JY, Choi YJ, et al. A practical MRI grading system for osteoarthritis of the knee: association with Kellgren-Lawrence radiographic scores. Eur J Radiol. 2013 Jan;82(1):112–7.

4. Schiphof D, Klerk BM de, Koes BW, Bierma-Zeinstra S. Good reliability, questionable validity of 25 different classification criteria of knee osteoarthritis: a systematic appraisal. J Clin Epidemiol. 2008 Dec 1;61(12):1205-1215.e2.

5. Hunter DJ, Zhang W, Conaghan PG, Hirko K, Menashe L, Reichmann WM, et al. Responsiveness and reliability of MRI in knee osteoarthritis: a meta-analysis of published evidence. Osteoarthr Cartil OARS Osteoarthr Res Soc. 2011 May;19(5):589–605.

6. A1CNow+ System – PTS Diagnostics [Internet]. [cited 2022 Sep 8]. Available from: https://ptsdiagnostics.com/a1cnow-plus-system/

7. Standards of Medical Care in Diabetes—2010. Diabetes Care. 2010 Jan;33(Suppl 1):S11–61.

8. Dehghan M, Merchant AT. Is bioelectrical impedance accurate for use in large epidemiological studies? Nutr J. 2008 Sep 9;7:26.

9. Tegtmeyer FK, Otte J, Horn C. [Determination of C-reactive protein (CRP) in capillary blood--a comparison with the CPR level in venous serum]. Monatsschrift Kinderheilkd Organ Dtsch Ges Kinderheilkd. 1990 Aug;138(8):443–5.

10. Lipovšek T, Kacin A, Puh U. Reliability and validity of hand-held dynamometry for assessing lower limb muscle strength. Isokinet Exerc Sci. 2022 Jan 1;30(3):231–40.

11. Baert IAC, Lluch E, Struyf T, Peeters G, Van Oosterwijck S, Tuynman J, et al. Inter- and intrarater reliability of two proprioception tests using clinical applicable measurement tools in subjects with and without knee osteoarthritis. Musculoskelet Sci Pract. 2018 Jun 1;35:105–9.

12. Roos EM, Lohmander LS. The Knee injury and Osteoarthritis Outcome Score (KOOS): from joint injury to osteoarthritis. Health Qual Life Outcomes. 2003 Nov 3;1:64.

13. Dell’Isola A, Allan R, Smith SL, Marreiros SS, Steultjens M. Identification of clinical phenotypes in knee osteoarthritis: a systematic review of the literature. BMC Musculoskelet Disord. 2016/10/14 ed. 2016 Oct 12;17(1):425.

14. Artus M, Campbell P, Mallen CD, Dunn KM, Windt DAW van der. Generic prognostic factors for musculoskeletal pain in primary care: a systematic review. BMJ Open. 2017 Jan 1;7(1):e012901.

15. Noble PC, Scuderi GR, Brekke AC, Sikorskii A, Benjamin JB, Lonner JH, et al. Development of a new Knee Society scoring system. Clin Orthop. 2012 Jan;470(1):20–32.

16. Gill S, Hely R, Page RS, Hely A, Harrison B, Landers S. Thirty second chair stand test: Test–retest reliability, agreement and minimum detectable change in people with early-stage knee osteoarthritis. Physiother Res Int. 2022;27(3):e1957.

17. Alghadir AH, Anwer S, Iqbal A, Iqbal ZA. Test-retest reliability, validity, and minimum detectable change of visual analog, numerical rating, and verbal rating scales for measurement of osteoarthritic knee pain. J Pain Res. 2018/05/08 ed. 2018;11:851–6.

18. Kregel J, Vuijk PJ, Descheemaeker F, Keizer D, van der Noord R, Nijs J, et al. The Dutch Central Sensitization Inventory (CSI): Factor Analysis, Discriminative Power, and Test-Retest Reliability. Clin J Pain [Internet]. 2016;32(7). Available from: https://journals.lww.com/clinicalpain/Fulltext/2016/07000/The_Dutch_Central_Sensitization_Inventory__CSI__.10.aspx

19. Novy D, Engle MP, Lai EA, Cook C, Martin EC, Trahan L, et al. Effectiveness of Splanchnic Nerve Neurolysis for Targeting Location of Cancer Pain: Using the Pain Drawing as an Outcome Variable. Pain Physician.

20. Baert IA, Lluch E, Mulder T, Nijs J, Noten S, Meeus M. Does pre-surgical central modulation of pain influence outcome after total knee replacement? A systematic review. Osteoarthritis Cartilage. 2015/09/19 ed. 2016 Feb;24(2):213–23.

21. Edwards RR, Dworkin RH, Turk DC, Angst MS, Dionne R, Freeman R, et al. Patient phenotyping in clinical trials of chronic pain treatments: IMMPACT recommendations. 2018;45.

22. Wylde V, Palmer S, Learmonth ID, Dieppe P. Test–retest reliability of Quantitative Sensory Testing in knee osteoarthritis and healthy participants. Osteoarthritis Cartilage. 2011 Jun 1;19(6):655–8.

23. Wessel J. The reliability and validity of pain threshold measurements in osteoarthritis of the knee. Scand J Rheumatol. 1995;24(4):238–42.

24. Penman ID, Ralston SH, Strachan MWJ, Hobson R. Davidson’s Principles and Practice of Medicine E-Book: Davidson’s Principles and Practice of Medicine E-Book. Elsevier Health Sciences; 2022. 1428 p.

25. Cathcart S, Winefield AH, Rolan P, Lushington K. Reliability of temporal summation and diffuse noxious inhibitory control. Pain Res Manag. 2009 Dec;14(6):433–8.

26. Dams L, Haenen V, Van der Gucht E, Devoogdt N, Smeets A, Bernar K, et al. Absolute and Relative Reliability of a Comprehensive Quantitative Sensory Testing Protocol in Women Treated for Breast Cancer. Pain Med. 2022 May 30;23(6):1162–75.

27. Ashoorion V, Sadeghirad B, Wang L, Noori A, Abdar M, Kim Y, et al. Predictors of persistent post-surgical pain following total knee arthroplasty: A systematic review and meta-analysis of observational studies. Pain Med. 2022 Oct 18;pnac154.

28. Osman A, Barrios FX, Gutierrez PM, Kopper BA, Merrifield T, Grittmann L. The Pain Catastrophizing Scale: Further Psychometric Evaluation with Adult Samples. J Behav Med. 2000 Aug 1;23(4):351–65.

29. Ong WJ, Kwan YH, Lim ZY, Thumboo J, Yeo SJ, Yeo W, et al. Measurement properties of Pain Catastrophizing Scale in patients with knee osteoarthritis. Clin Rheumatol. 2021 Jan;40(1):295–301.

30. Spinhoven P, Ormel J, Sloekers PP, Kempen GI, Speckens AE, Van Hemert AM. A validation study of the Hospital Anxiety and Depression Scale (HADS) in different groups of Dutch subjects. Psychol Med. 1997/03/01 ed. 1997 Mar;27(2):363–70.

31. Leysen M, Nijs J, Meeus M, Paul van Wilgen C, Struyf F, Vermandel A, et al. Clinimetric properties of illness perception questionnaire revised (IPQ-R) and brief illness perception questionnaire (Brief IPQ) in patients with musculoskeletal disorders: A systematic review. Man Ther. 2015 Feb;20(1):10–7.

**Supplementary table S2: Demographics and number of missing values total KOA sample for AIM 2**

| **Variable** | **Mean +/- SD or N (%) (n=197)** | **N (%) missing** |
| --- | --- | --- |
| **Continuous variables** | | |
| Age | 65.35 +/- 7.67 | 0 |
| BMI (kg/m^2^) | 29.97 +/- 5.31 | 0 |
| PPT m. Tibialis anterior (Ne) | 50.06 +/- 23.45 | 0 |
| PPT medial knee joint-line (Ne) | 42.12 +/- 22.53 | 0 |
| PPT lateral knee joint-line (Ne) | 46.99 +/- 24.63 | 0 |
| PPT m. ECRL (Ne) | 37.30 +/- 17.15 | 0 |
| PPT forehead (Ne) | 30.17 +/- 12.73 | 23 (11.86) |
| TS medial knee joint-line (Diff in NRS) | 1.25 +/- 1.99 | 0 |
| After sensation medial knee joint-line (0-10) | 0.43 +/- 1.15 | 0 |
| TS medial wrist (Diff in NRS) | 1.06 +/- 1.63 | 0 |
| Cold allodynia medial knee joint-line (0-10) | 0.36 +/- 0.97 | 1 (0.51) |
| Heat allodynia medial knee joint-line (0-10) | 0.87 +/- 1.52 | 1 (0.51) |
| Cold allodynia lateral knee joint-line (0-10) | 0.30 +/- 0.96 | 1 (0.51) |
| Heat allodynia lateral knee joint-line (0-10) | 0.41 +/- 1.14 | 1 (0.51) |
| Cold allodynia m. ECRL (0-10) | 0.20 +/- 0.79 | 1 (0.51) |
| Heat allodynia m. ECRL (0-10) | 0.48 +/- 1.16 | 1 (0.51) |
| CPM relative score (%) | 14.39 +/- 66.14 | 15 (7.61) |
| Strength m. Quadriceps (kgf) | 27.52 +/- 12.99 | 0 |
| Strength m. Hamstrings (kgf) | 11.94 +/- 5.92 | 0 |
| Proprioception (°) | 4.47 +/- 2.09 | 3 (1.52) |
| 30s chair stand test (N) | 10.76 +/- 4.06 | 2 (1.02) |
| Hba1c value (%) | 5.58 +/- 0.59 | 16 (8.12) |
| Bodychart (N) | 3.47 +/- 2.27 | 0 |
| NRS pain in rest (0-10) | 4.60 +/- 2.67 | 0 |
| IPQR identitiy score (0-14) | 2.12 +/- 1.42 | 0 |
| IPQR Timeline (6-30) | 17.87 +/- 5.28 | 0 |
| IPQR Consequences (6-30) | 19.46 +/- 4.21 | 0 |
| IPQR personal control (6-30) | 19.70 +/- 3.98 | 0 |
| IPQR treatment control (5-25) | 18.16 +/- 3.08 | 0 |
| IPQR Illness cohorence (5-25) | 18.74 +/- 2.14 | 0 |
| IPQR Timeline cyclical (4-20) | 11.95 +/- 3.84 | 0 |
| IPQR Emotional representations (6-30) | 15.80 +/- 4.58 | 0 |
| PCS rumination (0-16) | 6.26 +/- 3.84 | 1 (0.51) |
| PCS magnification (0-12) | 2.73 +/- 2.52 | 1 (0.51) |
| PCS helplesness (0-24) | 7.36 +/- 5.08 | 1 (0.51) |
| PCS total score (0-52) | 16.35 +/- 10.41 | 1 (0.51) |
| HADS fear (0-21) | 5.31 +/- 3.94 | 0 |
| HADS depression (0-21) | 5.07 +/- 3.20 | 0 |
| KSSS symptoms (0-20) | 8.46 +/- 4.67 | 0 |
| KSSS satisfaction (0-40) | 15.40 +/- 7.31 | 1 (0.51) |
| KSSS expectations (3-15) | 13.95 +/- 1.61 | 1 (0.51) |
| KSSS functional score (0-100) | 43.14 +/- 15.13 | 1 (0.51) |
| KOOS subscale symptoms (0-20) | 48.85 +/- 17.82 | 1 (0.51) |
| KOOS subscale pain (0-100) | 43.82 +/- 15.30 | 1 (0.51) |
| CSI (0-100) | 28.23 +/- 12.99 | 1 (0.51) |
| Fat mass (%) | 35.04 +/- 8.92 | 83 (42.13) |
| Lean mass (%) | 64.96 +/- 8.92 | 83 (42.13) |
| CRP-value (mg/L) | 6.75 +/- 20.12 | 109 (55.33) |
| KOOS subscale pain one-year postoperative | 72.76 +/- 24.57 | 41 (20.80) |
| **Categorical variables** | | |
| Sex Man | 102 (52) | 0 |
| Woman | 95 (48) |  |
| Grade of KOA K&L 1 | 3 (1.50) | 6 (3.00) |
| K&L 2 | 40 (20.30) |  |
| K&L 3 | 68 (34.50) |  |
| K&L 4 | 80 (40.60) |  |
| Education No degree | 11 (5.60) | 0 |
| Primary school | 11 (5.60) |  |
| Technical secondary school | 46 (23.40) |  |
| Higher secondary school | 24 (12.20) |  |
| High school | 48 (24.37) |  |
| University | 16 (8.10) |  |
| Other | 41 (20.80) |  |
| Work Pension | 104 (52.80) | 1 (0.50) |
| Self-employed | 14 (7.10) |  |
| White-collar worker | 26 (13.20) |  |
| Laborer | 25 (12.70) |  |
| Unemployed | 2 (1.00) |  |
| Other | 25 (12.70) |  |
| Marital status Married | 141 (71.60) | 1 (0.50) |
| Divorced | 17 (8.60) |  |
| Single | 8 (4.10) |  |
| Widow(er) | 18 (9.10) |  |
| Other | 12 (6.10) |  |
| Abbreviations: SD= standard deviation, N= number, BMI= body mass index, kg/m^2^= kilograms/squared meter, PPT= pressure pain threshold, m. = musculus, Ne= Newton, ECRL= extensor carpi radialis longus, TS= temporal summation, Diff= difference, NRS= numeric rating scale, CPM= conditioned pain modulation, kgf= kilograms force, Hb1ac= glycated hemoglobin, IPQR= illness perceptions questionnaire revised, PCS= pain catastrophizing scale, HADS= hospitality anxiety and depression scale, KSSS= knee society scoring system, KOOS= knee injury and osteoarthritis outcome scale, CSI= central sensitization inventory, CRP= C-reactive protein, K&L= Kellgren and Lawrence scale | | |

**Supplementary Table S3: Differences between knee osteoarthritis participants with ‘probable’, ‘possible’, or ‘no’ nociplastic pain (continuous variables) at baseline and one-year postoperative (4 pain locations approach)**

| **Variable** | **Probable nociplastic pain (n = 30)** | **Possible nociplastic pain (n= 20)** | **No nociplastic pain (n= 147)** | **P-value** | **Post-hoc** |
| --- | --- | --- | --- | --- | --- |
| **Continuous variables** | **Estimated mean (95%CI)** | | |  |  |
| **Demographic variable** | | | | |  |
| Age | 61.83 (59.14; 64.53) | 64.70 (61.39; 68.01) | 66.16 (64.94; 67.38) | 0.017 | / |
| **Metabolic and inflammatory variables** | | | | |  |
| BMI (kg/m^2^) | 29.85 (27.90; 31.79) | 28.45 (26.13, 30.77) | 30.21 (29.35; 31.08) | 0.380 | / |
| Hba1c value (%) | 5.64 (5.40; 5.88) | 5.48 (5.21; 5.76) | 5.59 (5.48; 5.69) | 0.634 | / |
| **Pain-related variables** | | | | |  |
| Bodychart (N) | 6.46 (5.83; 7.09) | 5.08 (4.33; 5.83) | 2.65 (2.37; 2.93) | <0.001* | Probable vs. no + possible vs. no : <0.001*, probable vs. possible: 0.019* |
| NRS pain in rest (0-10) | 4.88 (3.90; 5.56) | 5.38 (4.21; 6.55) | 4.46 (4.02; 4.89) | 0.308 | / |
| KOOS subscale pain (0-100) | 38.09 (32.60; 43.57) | 44.84 (38.29; 51.38) | 44.89 (42.44; 47.34) | 0.088 | / |
| PPT m. Tibialis anterior (Ne) | 42.65 (34.98; 50.31) | 47.56 (38.41; 56.70) | 51.46 (48.04; 54.87) | 0.118 | / |
| PPT MK joint-line (Ne) | 30.91 (23.16; 38.65) | 38.70 (29.46; 47.94) | 44.59 (41.15; 48.04) | 0.007* | Probable vs. no: 0.006* |
| PPT LK joint-line (Ne) | 36.49 (28.06; 44.92) | 44.74 (34.68; 54.80) | 49.35 (45.60; 53.10) | 0.026 | / |
| PPT m. ECRL (Ne) | 30.73 (25.12; 36.34) | 35.41 (28.72; 42.10) | 38.33 (35.84; 40.83) | 0.055 | / |
| PPT forehead (Ne) | 25.00 (20.42; 29.59) | 28.32 (22.64; 34.01) | 31.40 (29.29; 33.52) | 0.038 | / |
| TS MK joint-line (Diff in NRS) | 2.24 (1.55; 2.93) | 2.22 (1.40; 3.04) | 0.94 (0.63; 1.24) | <0.001* | Probable vs. no: 0.003*, possible vs. no: 0.014* |
| After sensation medial knee (0-10) | 1.15 (0.75; 1.54) | 1.12 (0.65; 1.59) | 0.19 (0.02; 0.37) | <0.001* | Probable vs. no: <0.001*, possible vs. no: 0.001* |
| TS medial wrist (Diff in NRS) | 1.83 (1.24; 2.42) | 1.36 (0.66; 2.06) | 0.86 (0.60; 1.13) | 0.012* | Probable vs. no: 0.012* |
| After sensation medial wrist (0-10) | 0.27 (0.04; 0.50) | 0.44 (0.17; 0.71) | 0.11 (0.01; 0.21) | 0.062 | / |
| Cold allodynia MK joint-line (0-10) | 1.04 (0.70; 1.39) | 0.56 (0.15; 0.97) | 0.20 (0.05; 0.36) | <0.001* | Probable vs. no: <0.001* |
| Heat allodynia MK joint-line (0-10) | 1.87 (1.32; 2.41) | 1.28 (0.64; 1.93) | 0.62 (0.38; 0.87) | <0.001* | Probable vs. no: <0.001* |
| Cold allodynia LK joint-line (0-10) | 0.96 (0.61; 1.30) | 0.30 (-0.11; 0.71) | 0.17 (0.02; 0.32) | <0.001* | Probable vs. no: <0.001*, probable vs. possible: 0.046* |
| Heat allodynia LK joint-line (0-10) | 1.22 (0.80; 1.63) | 0.53 (0.03; 1.02) | 0.24 (0.05; 0.43) | <0.001* | Probable vs. no: <0.001* |
| Cold allodynia m. ECRL (0-10) | 0.51 (0.22; 0.81) | 0.15 (-0.20; 0.50) | 0.15 (0.02; 0.29) | 0.093 | / |
| Heat allodynia m. ECRL (0-10) | 1.09 (0.67; 1.52) | 0.83 (0.33; 1.34) | 0.33 (0.14; 0.52) | 0.003* | Probable vs. no: 0.005* |
| CPM relative score (%) | 7.89 (-16.49; 32.26) | 43.02 (12.93; 73.12) | 12.61 (1.40; 23.82) | 0.133 | / |
| CSI (0-100) | 40.30 (36.11; 44.49) | 26.47 (21.47; 31.47) | 23.30 (24.44; 28.17) | <0.001* | Probable vs. no + probable vs. possible : <0.001* |
| **Functional variables** | | | | |  |
| Strength m. Quadriceps (kgf) | 23.56 (19.62; 27.49) | 27.39 (22.70; 32.09) | 28.02 (26.27; 29.77) | 0.137 | / |
| Strength m. Hamstrings (kgf) | 9.95 (8.00; 11.90) | 10.15 (7.73; 12.48) | 12.48 (11.31; 13.34) | 0.027 | / |
| Proprioception (°) | 4.30 (3.52; 5.08) | 5.05 (4.14; 5.97) | 4.41 (4.07; 4.76) | 0.370 | / |
| 30s chair stand test (N) | 9.86 (8.37; 11.35) | 10.39 (8.62; 12.17) | 10.98 (10.32; 11.64) | 0.380 | / |
| KSSS symptoms (0-20) | 8.10 (6.43; 9.77) | 7.60 (5.61; 9.60) | 8.63 (7.89; 9.38) | 0.590 | / |
| KSSS functional score (0-100) | 37.58 (32.15; 43.02) | 43.46 (36.98; 49.94) | 44.09 (41.67; 46.51) | 0.106 | / |
| KOOS subscale symptoms (0-100) | 9.87 (8.60; 11.14) | 10.21 (8.70; 11.72) | 10.32 (9.76; 10.89) | 0.817 | / |
| **Psychological variables** | | | | |  |
| IPQR identity score (0-14) | 2.21 (1.69; 2.73) | 2.15 (1.53; 2.77) | 2.10 (1.87; 2.34) | 0.933 | / |
| IPQR Timeline (6-30) | 19.04 (17.09; 20.99) | 18.39 (16.07; 20.72) | 17.54 (16.68; 18.41) | 0.363 | / |
| IPQR Consequences (6-30) | 19.33 (17.79; 20.87) | 19.41 (17.57; 21.24) | 19.47 (18.79; 20.16) | 0.986 | / |
| IPQR personal control (6-30) | 19.82 (18.35; 21.29) | 20.29 (18.53; 22.04) | 19.59 (18.93; 20.24) | 0.757 | / |
| IPQR treatment control (5-25) | 17.86 (16.72; 18.99) | 18.52 17.26; 19.97) | 18.15 (17.65; 18.66) | 0.700 | / |
| IPQR Illness cohorence (5-25) | 19.31 (18.53; 20.09) | 18.39 (17.46; 19.33) | 18.66 (18.31; 19.01) | 0.256 | / |
| IPQR Timeline cyclical (4-20) | 11.92 (10.50; 13.34) | 12.49 (10.80; 14.19) | 11.98 (11.26; 12.52) | 0.808 | / |
| IPQR Emotional representations (6-30) | 17.61 (15.96; 19.26) | 14.73 (12.76; 16.70) | 15.60 (14.86; 16.33) | 0.051 | / |
| PCS rumination (0-16) | 7.42 (6.01; 8.82) | 5.58 (3.82; 7.34) | 6.12 (5.49; 6.74) | 0.177 | / |
| PCS magnification (0-12) | 3.70 (2.78; 4.62) | 3.25 (2.13; 4.37) | 2.45 (2.04; 2.86) | 0.039 | / |
| PCS helplesness (0-24) | 9.10 (7.26; 10.45) | 7.31 (5.07; 9.56) | 7.01 (6.19; 7.83) | 0.137 | / |
| PCS total score (0-52) | 20.22 (16.44; 24.00) | 16.14 (11.54; 20.74) | 15.58 (13.90; 17.26) | 0.097 | / |
| HADS fear (0-21) | 7.13 (5.73; 8.52) | 6.15 (4.49; 7.81) | 4.85 (4.23; 5.47) | 0.011* | Probable vs. no: 0.013* |
| HADS depression (0-21) | 6.88 (5.73; 8.04) | 5.10 (3.73; 6.48) | 4.69 (4.18; 5.20) | 0.004* | Probable vs. no: 0.003* |
| KSSS satisfaction (0-40) | 12.95 (10.32; 15.57) | 14.46 (11.33; 17.60) | 15.99 (14.81; 17.16) | 0.110 | / |
| KSSS expectations (3-15) | 13.48 (12.89; 14.07) | 14.18 (13.42; 14.83) | 14.02 (13.76; 14.29) | 0.234 | / |
| **One year postoperative outcome variable** | | | | |  |
| KOOS subscale pain | 58.56 (48.41; 68.71) | 70.75 (58.39; 83.11) | 75.09 (70.66; 79.52) | 0.005* | Probable vs. no: 0.004* |
| Table 4. Blue* is significant difference (p<0.017). All variables are adjusted for sex and age (except age itself). Abbreviations: BMI= body mass index. kg/m2= kilograms/squared meter. PPT= pressure pain threshold. m. = musculus. Ne= Newton. ECRL= extensor carpi radialis longus. TS= temporal summation. Diff= difference. NRS= numeric rating scale. CPM= conditioned pain modulation. kgf= kilograms force. Hb1ac= glycated hemoglobin. IPQR= illness perceptions questionnaire revised. PCS= pain catastrophizing scale. HADS= hospitality anxiety and depression scale. KSSS= knee society scoring system. KOOS= knee injury and osteoarthritis outcome scale. CSI= central sensitization inventory, MK= medial knee, LK= lateral knee. | | | | | |

**Supplementary Table S4: Differences between knee osteoarthritis participants with ‘probable’, ‘possible’, or ‘no’ nociplastic pain (categorical variables) at baseline and one-year postoperative (4 pain locations approach)**

| **Variable** | **Probable nociplastic pain (n = 30)** | **Possible nociplastic pain (n = 20)** | | **No nociplastic pain (n = 147)** | **P-value** | **Post-hoc** |
| --- | --- | --- | --- | --- | --- | --- |
| **Categorical variables** | **N (%)** | | | |  |  |
| **Demographic variable** |  | | |  |  |  |
| Sex Man | 9 (30.00) | | 8 (40.00) | 85 (57.82) | 0.011* | Probable vs. no: 0.005* |
| Woman | 21 (70.00) | | 12 (60.00) | 62 (42.18) |  |  |
| **Structural variable** | | | | | |  |
| Grade of KOA K&L 1 | 1 (3.33) | 0 (0.00) | | 2 (0.01) | 0.527 | / |
| K&L 2 | 10 (33.33) | 4 (20.00) | | 28 (19.05) |  |  |
| K&L 3 | 10 (33.33) | 5 (25.00) | | 55 (37.41) |  |  |
| K&L 4 | 9 (30.00) | 11 (55.00) | | 62 (42.18) |  |  |
| **Social variables** | | | | | |  |
| Education No degree | 3 (10.00) | 1 (5.00) | | 7 (4.76) | 0.757 | / |
| Primary school | 1 (3.33) | 2 (10.00) | | 8 (5.44) |  |  |
| Technical secondary school | 5 (16.67) | 3 (15.00) | | 38 (25.85) |  |  |
| Higher secondary school | 3 (10.00) | 3 (15.00) | | 19 (12.93) |  |  |
| High school | 9 (30.00) | 8 (40.00) | | 30 (20.41) |  |  |
| University | 3 (10.00) | 0 (0.00) | | 13 (8.84) |  |  |
| Other | 6 (20.00) | 3 (15.00) | | 32 (21.77) |  |  |
| Work Pension | 9 (30.00) | 10 (50.00) | | 86 (58.50) | 0.296 | / |
| Self-employed | 5 (16.67) | 4 (20.00) | | 5 (3.40) |  |  |
| White-collar worker | 6 (20.00) | 2 (10.00) | | 18 (12.24) |  |  |
| Laborer | 4 (13.33) | 2 (10.00) | | 19 (12.93) |  |  |
| Unemployed | 0 (0.00) | 0 (0.00) | | 2 (1.36) |  |  |
| Other | 6 (20.00) | 2 (10.00) | | 17 (11.56) |  |  |
| Marital status Married | 20 (66.67) | 15 (75.00) | | 107 (72.79) | 0.465 | / |
| Divorced | 3 (10.00) | 2 (10.00) | | 12 (8.16) |  |  |
| Single | 3 (10.00) | 0 (0.00) | | 5 (3.40) |  |  |
| Widow(er) | 1 (3.33) | 3 (15.00) | | 14 (9.52) |  |  |
| Other | 3 (10.00) | 0 (0.00) | | 9 (6.12) |  |  |
| Table 5. Blue* is significant difference (p<0.017). All variables are adjusted for age and sex (except sex itself). Abbreviations: K&L= Kellgren and Lawrence scale | | | | | | |

**Supplementary Table S5: Differences between knee osteoarthritis participants with ‘probable’, ‘possible’, or ‘no’ nociplastic pain (continuous variables) at baseline and one-year postoperative (3 pain locations approach)**

| **Variable** | **Probable nociplastic pain (n = 46)** | **Possible nociplastic pain (n= 36)** | **No nociplastic pain (n= 115)** | **P-value** | **Post-hoc** |
| --- | --- | --- | --- | --- | --- |
| **Continuous variables** | **Estimated mean (95%CI)** | | |  |  |
| **Demographic variable** | | | | |  |
| Age | 62.41 (60.25; 64.58) | 64.58 (62.14; 67.03) | 66.77 (65.40; 68.13) | 0.004* | Probable vs. no: 0.003* |
| **Metabolic and inflammatory variables** | | | | |  |
| BMI (kg/m^2^) | 29.84 (28.28; 31.41) | 28.55 (26.83; 30.27) | 30.48 (29.50; 31.46) | 0.165 | / |
| Hba1c value (%) | 5.65 (5.46; 5.84) | 5.42 (5.22; 5.63) | 5.61 (5.49; 5.72) | 0.165 | / |
| **Pain-related variables** | | | | |  |
| Bodychart (N) | 5.24 (4.67; 5.81) | 4.26 (3.63; 4.89) | 2.53 (2.17; 2.89) | <0.001* | Probable vs. no + possible vs. no: <0.001* |
| NRS pain in rest (0-10) | 5.10 (4.31; 5.88) | 5.17 (4.31; 6.03) | 4.25 (3.75; 4.74) | 0.086 | / |
| KOOS subscale pain (0-100) | 39.07 (34.65; 43.49) | 44.00 (39.14; 48.86) | 45.72 (42.93; 48.51) | 0.053 |  |
| PPT m. Tibialis anterior (Ne) | 46.82 (39.92; 52.34) | 46.82 (39.99; 53.65) | 52.05 (48.15; 55.96) | 0.206 | / |
| PPT MK joint-line (Ne) | 33.36 (27.12; 39.61) | 40.01 (33.14; 46.87) | 45.93 (42.00; 49.85) | 0.005* | Probable vs. no: 0.004* |
| PPT LK joint-line (Ne) | 40.92 (34.08; 47.75) | 43.44 (35.93; 50.96) | 50.41 (46.11; 54.70) | 0.052 | / |
| PPT m. ECRL (Ne) | 32.64 (28.12; 37.16) | 34.56 (29.59; 39.54) | 39.30 (36.46; 42.14) | 0.038 | / |
| PPT forehead (Ne) | 27.74 (23.95; 31.53) | 29.34 (25.01; 33.68) | 31.30 (28.92; 33.68) | 0.265 | / |
| TS MK joint-line (Diff in NRS) | 1.78 (1.22; 2.35) | 1.78 (1.16; 2.41) | 0.90 (0.54; 1.25) | 0.010* | Probable vs. no: 0.036* |
| After sensation medial knee (0-10) | 0.75 (0.42; 1.08) | 0.74 (0.38; 1.10) | 0.21 (0.00; 0.42) | 0.007* | Probable vs. no: 0.027*, possible vs. no: 0.043* |
| TS medial wrist (Diff in NRS) | 1.63 (1.16; 2.11) | 1.13 (0.61; 1.65) | 0.81 (0.51; 1.11) | 0.021 | / |
| After sensation medial wrist (0-10) | 0.19 (0.00; 0.37) | 0.24 (0.04; 0.45) | 0.14 (0.02; 0.26) | 0.679 | / |
| Cold allodynia MK joint-line (0-10) | 0.78 (0.50; 1.07) | 0.39 (0.08; 0.70) | 0.19 (0.01; 0.38) | 0.004* | Probable vs. no: 0.003* |
| Heat allodynia MK joint-line (0-10) | 1.53 (1.09; 1.97) | 1.18 (0.69; 1.66) | 0.53 (0.25; 0.81) | <0.001* | Probable vs. no: <0.001* |
| Cold allodynia LK joint-line (0-10) | 0.61 (0.32; 0.90) | 0.27 (-0.04; 0.59) | 0.19 (0.01; 0.37) | 0.053 | / |
| Heat allodynia LK joint-line (0-10) | 0.78 (0.44; 1.13) | 0.42 (0.04; 0.80) | 0.27 (0.05; 0.49) | 0.050 | / |
| Cold allodynia m. ECRL (0-10) | 0.35 (0.11; 0.59) | 0.22 (-0.4; 0.48) | 0.15 (-0.01; 0.30) | 0.388 | / |
| Heat allodynia m. ECRL (0-10) | 0.85 (0.50; 1.20) | 0.68 (0.30; 1.06) | 0.30 (0.08; 0.51) | 0.019 | / |
| CPM relative score (%) | 4.77 (-14.97; 24.51) | 34.26 (12.22; 56.30) | 13.07 (0.25; 25.89) | 0.124 | / |
| CSI (0-100) | 38.78 (35.49; 42.07) | 24.38 (20.77; 28.00) | 25.59 (23.52; 27.66) | <0.001* | Probable vs. no + probable vs. possible: <0.001* |
| **Functional variables** | | | | |  |
| Strength m. Quadriceps (kgf) | 22.23 (19.12; 25.33) | 27.35 (23.94; 30.77) | 29.29 (27.34; 31.24) | 0.001* | Probable vs. no: <0.001* |
| Strength m. Hamstrings (kgf) | 10.44 (8.86; 12.02) | 10.97 (9.24; 12.71) | 12.70 (11.71; 13.69) | 0.041 | / |
| Proprioception (°) | 4.54 (3.91; 5.16) | 4.73 (4.05; 5.41) | 4.35 (3.96; 4.74) | 0.605 | / |
| 30s chair stand test (N) | 9.45 (8.26; 10.64) | 10.80 (9.50; 12.11) | 11.16 (10.51; 12.00) | 0.049 | / |
| KSSS symptoms (0-20) | 8.06 (6.71; 9.41) | 8.24 (6.76; 9.72) | 8.67 (7.82; 9.51) | 0.738 | / |
| KSSS functional score (0-100) | 37.12 (32.81; 41.48) | 43.43 (38.67; 48.20) | 45.28 (42.55; 48.01) | 0.010* | Probable vs. no: 0.007* |
| KOOS subscale symptoms (0-100) | 9.99 (8.97; 11.01) | 10.35 (9.22; 11.47) | 10.32 (9.67; 10.96) | 0.856 | / |
| **Psychological variables** | | | | |  |
| IPQR identity score (0-14) | 2.49 (2.07; 2.90) | 1.87 (1.42; 2.33) | 2.06 (1.80; 2.32) | 0.118 | / |
| IPQR Timeline (6-30) | 19.47 (17.90; 21.03) | 17.54 (15.82; 19.26) | 17.31 (16.33; 18.29) | 0.077 | / |
| IPQR Consequences (6-30) | 20.63 (19.40; 21.85) | 18.85 (17.50; 20.20) | 19.15 (18.38; 19.92) | 0.095 | / |
| IPQR personal control (6-30) | 19.63 (18.45; 20.82) | 20.22 (18.91; 21.53) | 19.55 (18.80; 20.30) | 0.682 | / |
| IPQR treatment control (5-25) | 17.69 (16.78; 18.60) | 18.87 (17.86; 19.87) | 18.12 (17.55; 18.69) | 0.277 | / |
| IPQR Illness cohorence (5-25) | 19.21 (18.58; 19.84) | 18.56 (17.86; 19.25) | 18.60 (18.20; 18.99) | 0.249 | / |
| IPQR Timeline cyclical (4-20) | 11.27 (10.13; 12.41) | 11.87 (10.61; 13.13) | 12.26 (11.55; 12.98) | 0.366 | / |
| IPQR Emotional representations (6-30) | 17.55 (16.23; 18.87) | 14.38 (12.93; 15.82) | 15.57 (14.74; 16.40) | 0.005* | Probable vs. no: 0.047*, probable vs. possible: 0.005* |
| PCS rumination (0-16) | 7.28 (6.15; 8.41) | 5.52 (4.24; 6.80) | 6.08 (5.37; 6.79) | 0.093 | / |
| PCS magnification (0-12) | 3.86 (3.13; 4.60) | 2.64 (1.82; 3.45) | 2.29 (1.83; 2.75) | 0.003* | Probable vs. no: 0.002* |
| PCS helplesness (0-24) | 9.53 (8.07; 10.99) | 6.86 (5.23; 8.49) | 6.65 (5.73; 7.57) | 0.005* | Probable vs. no: 0.005*, probable vs. possible: 0.043* |
| PCS total score (0-52) | 20.67 (17.66; 23.69) | 15.01 (11.66; 18.36) | 15.02 (13.13; 16.91) | 0.007* | Probable vs. no: 0.008*, probable vs. possible: 0.039* |
| HADS fear (0-21) | 6.67 (5.43; 7.80) | 5.16 (3.92; 6.40) | 4.85 (4.14; 5.56) | 0.032 | / |
| HADS depression (0-21) | 6.55 (5.62; 7.48) | 4.66 (3.64; 5.67) | 4.60 (4.02; 5.19) | 0.002* | Probable vs. no: 0.002*, probable vs. possible: 0.022* |
| KSSS satisfaction (0-40) | 13.64 (11.52; 15.77) | 14.97 (12.63; 17.31) | 16.18 (14.84; 17.53) | 0.148 | / |
| KSSS expectations (3-15) | 13.60 (13.12; 14.08) | 14.06 (13.53; 14.59) | 14.06 (13.75; 14.36) | 0.260 | / |
| **One year postoperative outcome variable** | | | | |  |
| KOOS subscale pain | 61.52 (53.81; 69.23) | 73.60 (54.42; 82.79) | 75.91 (70.72; 81.11) | 0.004* | Probable vs. no: 0.004* |
| Table 4. Blue* is significant difference (p<0.019). All variables are adjusted for sex and age (except age itself). Abbreviations: BMI= body mass index. kg/m2= kilograms/squared meter. PPT= pressure pain threshold. m. = musculus. Ne= Newton. ECRL= extensor carpi radialis longus. TS= temporal summation. Diff= difference. NRS= numeric rating scale. CPM= conditioned pain modulation. kgf= kilograms force. Hb1ac= glycated hemoglobin. IPQR= illness perceptions questionnaire revised. PCS= pain catastrophizing scale. HADS= hospitality anxiety and depression scale. KSSS= knee society scoring system. KOOS= knee injury and osteoarthritis outcome scale. CSI= central sensitization inventory, MK= medial knee, LK= lateral knee. | | | | | |

**Supplementary Table S6: Differences between knee osteoarthritis participants with ‘probable’, ‘possible’, or ‘no’ nociplastic pain (categorical variables) at baseline and one-year postoperative (3 pain locations approach)**

| **Variable** | **Probable nociplastic pain (n = 30)** | **Possible nociplastic pain (n = 36)** | | **No nociplastic pain (n = 115)** | **P-value** | **Post-hoc** |
| --- | --- | --- | --- | --- | --- | --- |
| **Categorical variables** | **N (%)** | | | |  |  |
| **Demographic variable** |  | | |  |  |  |
| Sex Man | 16 (34.78) | | 17 (47.22) | 46 (40.00) | 0.012* | Probable vs. no: 0.004* |
| Woman | 30 (65.22) | | 19 (52.78) | 69 (60.00) |  |  |
| **Structural variable** | | | | | |  |
| Grade of KOA K&L 1 | 3 (6.52) | 0 (0.00) | | 0 (0.00) | 0.035 | / |
| K&L 2 | 14 (30.43) | 5 (13.89) | | 23 (20.00) |  |  |
| K&L 3 | 13 (28.26) | 15 (41.67) | | 42 (36.52) |  |  |
| K&L 4 | 16 (34.78) | 16 (44.44) | | 50 (43.48) |  |  |
| **Social variables** | | | | | |  |
| Education No degree | 3 (6.52) | 1 (2.78) | | 7 (6.09) | 0.794 | / |
| Primary school | 2 (4.35) | 2 (5.56) | | 7 (6.09) |  |  |
| Technical secondary school | 13 (28.26) | 6 (16.67) | | 27 (23.48) |  |  |
| Higher secondary school | 5 (10.87) | 7 (19.44) | | 13 (11.30) |  |  |
| High school | 10 (21.74) | 10 (27.78) | | 27 (23.48) |  |  |
| University | 3 (6.52) | 1 (2.78) | | 12 (10.43) |  |  |
| Other | 10 (21.74) | 9 (25.00) | | 22 (19.13) |  |  |
| Work Pension | 16 (34.78) | 16 (44.44) | | 43 (37.39) | 0.051 | / |
| Self-employed | 6 (13.04) | 4 (11.11) | | 4 (3.48) |  |  |
| White-collar worker | 9 (19.57) | 4 (11.11) | | 13 (11.30) |  |  |
| Laborer | 6 (13.04) | 7 (19.44) | | 12 (10.43) |  |  |
| Unemployed | 0 (0.00) | 1 (2.78) | | 1 (0.01) |  |  |
| Other | 9 (19.57) | 4 (11.11) | | 12 (10.43) |  |  |
| Marital status Married | 32 (69.57) | 25 (69.44) | | 85 (73.91) | 0.660 | / |
| Divorced | 4 (8.70) | 4 (11.11) | | 9 (7.83) |  |  |
| Single | 3 (6.52) | 1 (2.78) | | 4 (3.48) |  |  |
| Widow(er) | 2 (4.35) | 5 (13.89) | | 11 (9.57) |  |  |
| Other | 5 (10.87) | 1 (2.78) | | 6 (5.22) |  |  |
| Table 5. Blue* is significant difference (p<0.017). All variables are adjusted for age and sex (except sex itself). Abbreviations: K&L= Kellgren and Lawrence scale | | | | | | |
